# Aquatic and riparian ecosystem recovery from debris flows in two western Washington streams, USA

**DOI:** 10.1002/ece3.5919

**Published:** 2020-02-24

**Authors:** Alex D. Foster, Shannon M. Claeson, Peter A. Bisson, John Heimburg

**Affiliations:** ^1^ USDA Forest Service Pacific Northwest Research Station Olympia Washington; ^2^ USDA Forest Service Pacific Northwest Research Station Wenatchee Washington; ^3^ Washington Department of Fish and Wildlife Olympia Washington

**Keywords:** barrier, colonization, dispersal, disturbance, landslide, riparian

## Abstract

An exceptionally powerful storm struck southwestern Washington in December 2007 causing large debris flows in two adjacent streams. The two affected streams had been studied prior to the storm, providing a rare opportunity to examine ecosystem recovery. We monitored the streams and their riparian zones for six years after the disturbances to determine whether recovery rates of biota, physical habitat, and water temperature differed, and if so, what factors affected resilience. Along both streams, the debris flows removed wide swaths of soil, rock, and coniferous riparian forests, widening the active channel and increasing solar exposure and summer water temperatures. Initially depauperate of vegetation, after four years red alder trees dominated the riparian plant communities. The warmer water, greater solar radiation, and unstable substrates likely contributed to variable benthic insect and tailed frog tadpole densities over time, although benthic insect communities became more similar after three years. The debris flows also decreased channel slopes and removed channel step barriers such that cutthroat trout were able to rapidly occupy habitats far upstream, but sculpins were slower to recolonize and both fish species exhibited some differences in recovery between the two streams. Crayfish were severely impacted by the debris flows; this may be due to attributes of their life history and the timing of the flows. Overall, we found that recolonizing aquatic species exhibited varying levels of resilience and recovery after the disturbances being related to the influence of physical habitat conditions, species dispersal ability, and the presence of nearby source populations.

## Graphical abstract

An exceptionally powerful storm caused large debris flows in two adjacent streams. The two affected streams had been studied prior to the storm providing a rare opportunity to examine ecosystem recovery. We monitored the streams and their riparian zones for 6 years after the disturbances to determine whether recovery rates of biota, physical habitat, and water temperature at each impacted stream differed, and if so, what factors affected resilience.

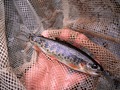

## 1 INTRODUCTION

In steep, headwater streams of the Pacific Northwest region, USA, saturated soils from high precipitation can trigger landslides and subsequent debris flows. Both landslides and debris flows are primary agents of change in aquatic ecosystems and dramatically affect channel and valley morphology, influencing aquatic and riparian habitats and associated biological communities (Benda, Hassan, Church, & May, 2005; Benda, Veldhuisen, & Black, 2003; Cover, de la Fuente, & Resh, 2010; Kiffney et al., 2004; Kirkby, 2013). Landslides often contribute a large pulse of hillslope sediment and large wood to stream channels. Driven by gravity, landslide material quickly mixes with water and accumulates additional substrate and wood from the stream channel and riparian area as it moves downstream. This forms a fast‐flowing slurry of fine and coarse sediment combined with trees and other organic debris (Takashi, 2014), often decimating biota in the stream and adjacent riparian area. However, debris‐flow delivery of coarse sediment and wood can increase wood‐associated pools known to be beneficial to salmonids (Benda et al., 2003; Reeves, Benda, Burnett, Bisson, & Sedell, 1995). As a result, the spacing and network pattern of debris‐flow‐prone headwater streams exhibits a high degree of physical heterogeneity (Bigelow, Benda, Miller, & Burnett, 2007; Borga, Stoffel, Marchi, Marra, & Jakob, 2014; Gomi, Sidle, & Swanston, 2004), and in this regard, debris flows are considered important agents of habitat formation in aquatic ecosystems (Bisson, Dunham, & Reeves, 2009).

Despite the long‐term aquatic habitat benefits of debris flows, the immediate consequences of these disturbances are usually catastrophic for biota in and adjacent to the impacted stream channels. Natural resource managers often undertake restoration actions such as restocking fish or creating habitat where structural roughness elements including large wood and boulders have been scoured (Montgomery et al., 2002). While the effects of restoration projects have received extensive study (Roni, 2005), fewer investigations have been conducted of streams that are recovering passively without intervention from both human‐caused and natural disturbances (Martens, Devine, Minkova, & Foster, 2019).

A powerful storm struck southwestern Washington State, USA, on 1–3 December 2007. Dubbed the “Great Coastal Gale,” this storm brought a combination of snow, gale force winds, and heavy rains which caused many landslides, debris flows, and severe lowland flooding across the region (Mote, Mault, & Duliere, 2007; Read, 2007). In Capitol State Forest, an actively managed forest near Olympia, Washington, two adjacent streams experienced debris flows during this December 2007 storm. Coincidentally, these two streams had several years of prestorm aquatic biota and temperature data, providing a rare opportunity to examine the effects of these unusual disturbances. Because of the unpredictable nature of debris flows, recovery studies typically involve comparisons between affected and unaffected areas, or retrospectively comparing changes at different intervals after an event. For example, Cover et al. (2010) used a space‐for‐time substitution on 10 debris‐flow‐affected streams in northern California to infer disturbance effects on stream ecosystem structure. In the Olympic Mountains of western Washington, periphyton biomass, water temperature, chemistry, and aquatic macroinvertebrates were compared between a debris‐flow‐affected stream and nearby control stream (Kiffney et al., 2004). Snyder and Johnson (2006) employed a study design that combined channel stratification along a debris‐flow track with a nearby stream that was not disturbed to assess differences in aquatic insect assemblages in Shenandoah National Park, Virginia.

To our knowledge, only three published debris‐flow studies were fortunate enough to have predisturbance data for before–after comparisons. In a western Oregon Cascade Range stream partially affected by a large debris flow, Lamberti, Gregory, Ashkenas, Wildman, and Moore (1991) compared an upstream unaffected area and downstream disturbed area. Roghair, Dolloff, and Underwood (2002) stratified a debris flow in Shenandoah National Park, Virginia (the same event studied by Snyder & Johnson, 2006), to assess brook trout (*Salvelinus fontinalis*) colonization and movements, comparing affected and unaffected areas. As cited previously, Kiffney et al. (2004) had predisturbance data and were able to make before–after comparisons for several metrics. Results are forthcoming from a fourth study conducted in a nearby watershed affected by the same 2007 storm event as our study (Fransen, Walter, Reiter, & Tarosky, 2013). In this study, we use predisturbance data for aquatic biota and water temperature for several years prior to the two adjacent debris flows to make before–after comparisons. The data were amassed as part of the Riparian Ecosystem Management Study (REMS) on headwater stream riparian buffers from 2002 to 2006 (Bisson, Claeson, Wondzell, Foster, & Steel, 2013).

The objectives of this study of debris flows were to (a) evaluate the recovery of the streams by fishes (primarily trout and sculpin), aquatic insects, and riparian vegetation; (b) document post‐debris flow changes in stream channel morphology and water temperatures; and (c) describe interactions among physical habitat, water temperature, and species colonization. Specifically, we wanted to determine whether the species patterns of temporal and spatial reoccupation were similar between the two disturbed streams, and how aquatic communities and habitat conditions compared before and after the debris flows and to a nearby unimpacted reference stream. We use the results of our monitoring to infer the mechanisms that underlie responses of biological communities in the post‐debris flow streams that we studied. These mechanisms include (a) sources of repopulating species, (b) recolonization rates of the species in the debris flows, (c) food web relationships among pioneer species, and (d) physical factors affecting colonization. The results can help inform management decisions about whether habitat restoration projects are needed in small stream ecosystems and whether restocking actions are necessary to accelerate the recovery of desired species such as salmonid fishes.

## 2 METHODS

### 2.1 Study sites

**Figure 1:**
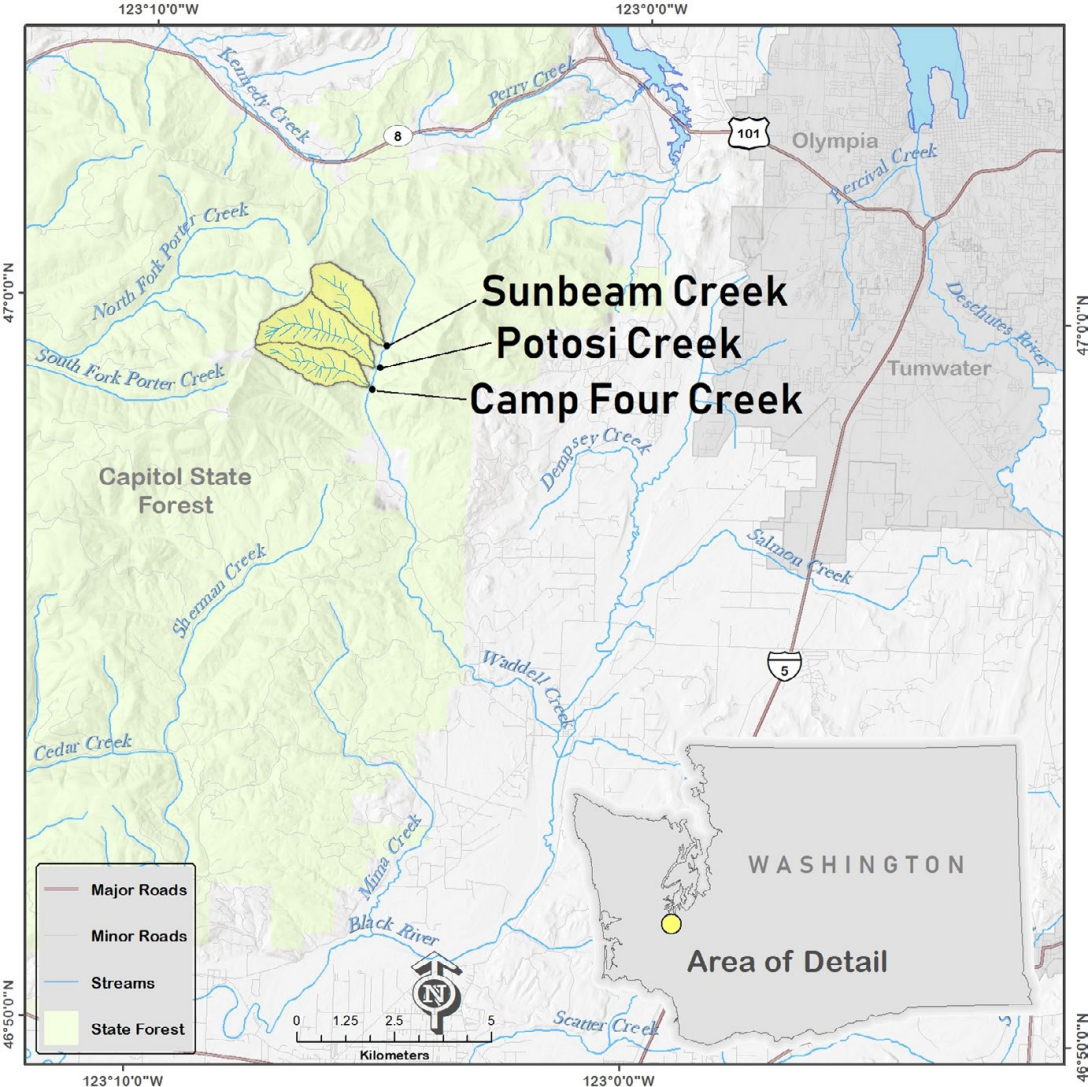
Study area location in Washington State, USA

Potosi, Camp Four, and Sunbeam creeks are three perennial, fish‐bearing streams whose watersheds are adjacent to each other in Capitol State Forest, an area managed by the Washington Department of Natural Resources (WADNR) located approximately 15 km west of the city of Olympia in Washington State, USA (Figure 1). Potosi and Camp Four creeks were disturbed by debris flows in December 2007, whereas Sunbeam Creek was not, although Sunbeam did experience high flows. All three streams are east‐facing 2nd‐ and 3rd‐order tributaries of Waddell Creek, which subsequently flows into the Black River, a major tributary of the Chehalis River in southwest Washington.

The Capitol State Forest lies in the Puget Trough physiographic province, located approximately 75 km from the Pacific Ocean, and receives on average 127 cm (*SD* = 22) of rainfall annually (source: COOP station # USW00024227, Olympia, WA; period of record: 1981–2010) (NCEI, 2019). Approximately 90% of rainfall occurs between October and April. Summers are typically dry with little rainfall from July to August. Bedrock lithology is predominately basalts of the Crescent Formation (Washington Division of Geology & Earth Resources, 2005). The watersheds of Potosi (2.76 km^2^), Camp Four (1.88 km^2^), and Sunbeam (2.00 km^2^) creeks are heavily forested. Land use in this area is primarily timber production and recreation; there are no rural developments or other human alterations in the watersheds of the study streams. Prior to the debris flows, the dominant tree species in Potosi, Camp Four, and Sunbeam riparian areas was second‐growth Douglas‐fir (*Pseudotsuga menziesii*) with an approximate age of 80 years. Using 1959 aerial photographs, the oldest we could access, there was no evidence of large landslides or debris flows in the Potosi, Camp Four, and Sunbeam watersheds.

**Figure 2:**
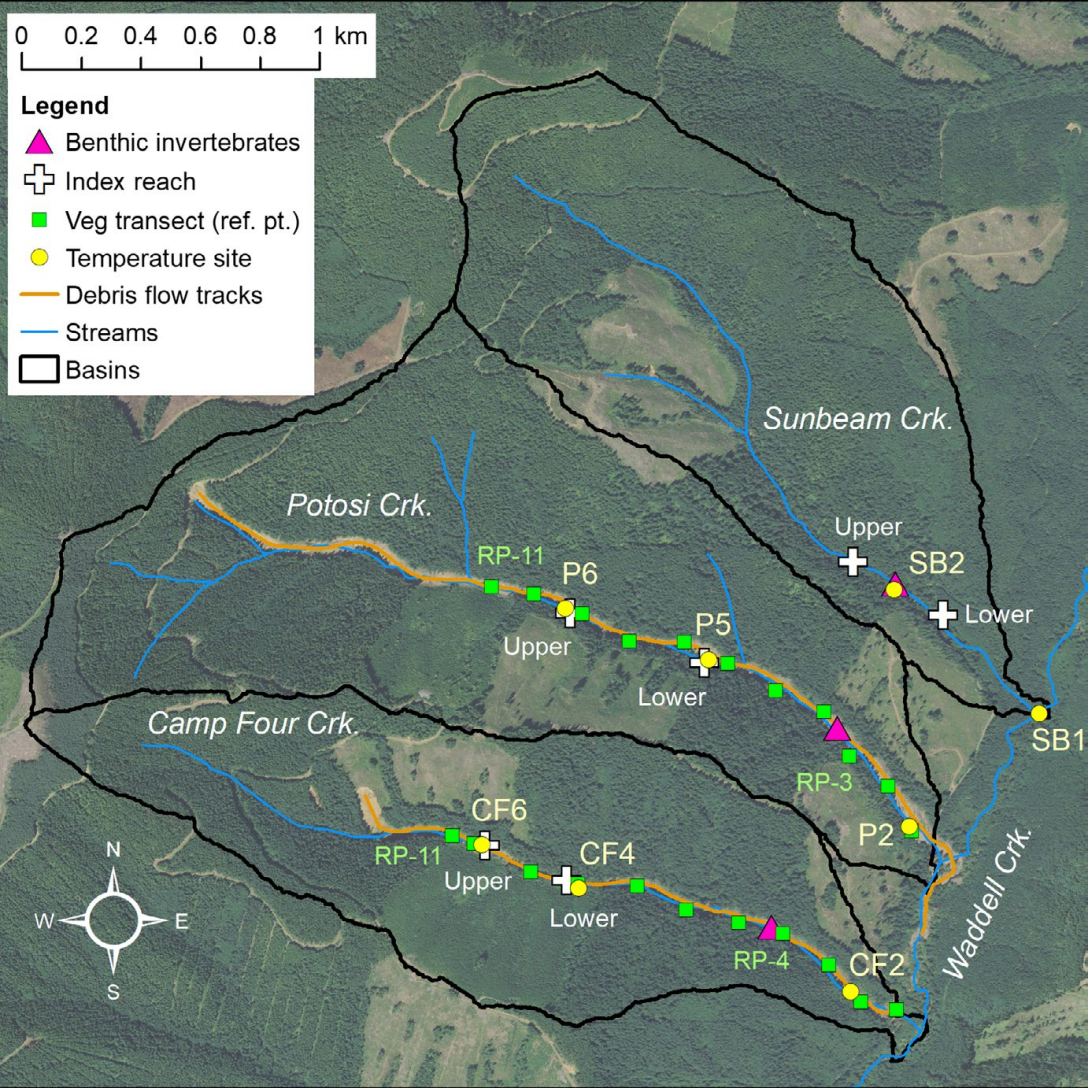
Debris‐flow sampling sites showing vegetation transects and reference points (RP), lower and upper index survey reaches, benthic invertebrate reaches, and temperature monitoring sites (SB, P, CF)

The 2007 Coastal Gale storm event was the proximate causal agent of the landslides and subsequent debris flows in Potosi and Camp Four creeks. Weather station data at Olympia 15 km east of the debris flows reported that 15 cm of rain fell during 1–3 December 2007 (~12% of the yearly average). The debris flow in Potosi Creek was initiated by fill failure of a side‐slope logging road located near the stream's headwall area (123.1236°W, 46.9966°N; elevation 518 m at the road failure). On adjacent Camp Four Creek, the debris flow originated from a shallow landslide in a 3.5‐year‐old clear‐cut (123.1154°W, 46.9876°N; elevation 395 m at the scarp; Figure 2). On Sunbeam Creek (123.0924°W, 46.9941°N; elevation 192 m at WADNR road C–8000), evidence of peak flow was observed (i.e., movement of boulders, large cobbles, wood; plus evidence of bank erosion), yet the stream did not experience a debris flow during the 2007 storm.

### 2.2 Sampling design

#### 2.2.1 Physical metrics

##### Channel morphology

To assess changes to channel morphology in Camp Four and Potosi creeks before (2002) and after (2011) the debris flows, we used Light Detection and Ranging (LiDAR) data. We first normalized the LiDAR data so that the same bare earth point density was obtained between data sets. We performed before and after impact comparisons for channel slope and valley floodplain width along both debris‐flow tracks. Channel‐slope profiles used x, y, and z cell values from the LiDAR data, computing an overall slope % from the elevation (z) change from the toe of the initiating landslide to the Waddell Creek confluence. Floodplain width was the distance perpendicular to the channel between the bases of opposing valley walls (Grant & Swanson, 1995; May, 2002) taken at specific locations coinciding with vegetation reference points (RPs) described below, as defined by high‐resolution contour maps derived from the LiDAR data. At each cross‐sectional location, we computed the difference and percent change in floodplain width before and after the debris flows.

##### Stream temperature

We collected stream temperatures in the summer for three years before (2003–2005) and six years after (2008–2013) the debris flows. In the predisturbance years, as part of the REMS study, we placed iButton^®^ (Maxim Integrated, precision of ±0.5°C) temperature loggers at three locations in Camp Four (CF2, CF4, and CF6), three in Potosi (P2, P5, and P6), and two in Sunbeam (SB1 and SB2) (Figure 2). After the debris flows, we again placed Tidbit^®^ (Onset, precision of ±0.2°C) temperature loggers at these same locations on Camp Four, Potosi, and Sunbeam creeks. Hourly water temperatures were recorded from 2 July to 23 September (84 days) for all of the years, except at CF2 in 2003 and CF6 and P5 in 2008 when the data loggers were compromised.

For each site and from the years 2003–2005 (pre‐debris flows) and 2008–2013 (post‐debris flows), we calculated summer water temperature (°C) response metrics of daily maximum and daily minimum. Other metrics reported include the summer maximum of the 7‐day average of the daily maxima (7DADM), and the number of days over 16.0°C and 23.0°C (incipient sublethal and lethal temperatures, respectfully, for salmon and trout juvenile rearing, EPA, 2003).

We calculated the daily maximum temperature difference for each debris‐flow site as compared to a control site for each year of the study, and repeated this step for daily minimum temperature differences. The site SB1 was used as the control for sites CF2 and P2, as they are all near the mouth of their respective streams, whereas SB2 was used as the control for CF4, CF6, P5, and P6, as they are all farther upstream (Figure 2). In the pre‐disturbance years, temperature response differences were most often zero between the disturbed and the control sites. Given the small variation in temperature differences within each 3‐month annual period of study, the temperature responses were modeled as the difference between disturbed site and control site temperature per day (i.e., *∆T = Ttrt_i_* − *Tref_i_* on each study day *i*). We use repeated‐measures ANOVA (R v.3.1.3) to test for significant differences in daily maximum and minimum temperature differences for each disturbed versus control site comparison between pre‐ and post years. If the ANOVA was significant, we used Dunnett's contrasts to compare each of the six postyear differences to a single control (all preyears combined). Dunnett's *t* test is designed to hold the familywise error rate at or below α when performing multiple comparisons of a number of treatments with a single control. Any pre‐existing differences between the sites prior to the debris flows were incorporated in the ANOVA mean estimate results. The ANOVA was repeated for each debris‐flow site and response metric (daily maximum, daily minimum). Note that water temperature data were not available in 2008 at the debris flow sites CF6 and P5.

#### 2.2.2 Biological metrics

##### Riparian vegetation

Surveys commenced in 2009 to assess vegetation recolonization and growth along the longitudinal profiles of Camp Four and Potosi creeks. We established two 5‐m‐diameter circular plots (19.6 m^2^) at equal distance along transects across the width of each debris flow. Starting from the confluence with Waddell Creek, plot transects occurred every 200 m going upstream (Figure 2). We GPS‐mapped the right‐bank terminus of each transect with differential correction. These locations were used as reference points (RPs) for spatial verification of other study components (e.g., fish distribution). We collected riparian vegetation data on 11 transects (22 plots) on Potosi Creek and 9 transects (18 plots) on Camp Four Creek. From 2009 to 2013 (Table 1), we sampled plots for vegetation once per year at the end of the growing season, typically late September (total of 200 plots sampled over five years).

**Table 1:** Sampling design summary

Metric	Method	Sites	Location at sites	Frequency	Sampling periods
Aquatic vertebrates and crayfish	Electrofishing, three‐pass removal with block nets	Potosi, Camp Four, Sunbeam (reference)	Two ~25‐m reaches spaced 500 m apart; 200 m apart on Sunbeam	Two surveys per year, July and September	Before debris flow 2002–2006 (Potosi), 2003–2006 (Camp Four); after debris flow 2008–2012 (Potosi, Camp Four, Sunbeam)
Aquatic insects	Surber sampler (500 μm mesh, 0.09 m^2^). Ten samples along 50 m of channel	Potosi, Camp Four, Sunbeam (reference)	One 50‐m reach, ~500 m upstream from Waddell	One survey per year, mid to late June	After debris flow 2008–2012
Fish distribution	Electrofishing. Trout: 400 m after last detection to confirm; sculpin: 200 m after last detection to confirm	Potosi, Camp Four, trout only in Sunbeam (reference)	Start at confirmed location from last survey	Two surveys per year, July and September	After debris flow 2008–2012
Riparian vegetation	Two 5‐m circular plots (19.6 m^2^) on transect across debris‐flow track	Potosi, Camp Four	Transects every 200 m along debris flows	One survey per year, late September	After debris flow 2009–2013
Stream temperature	iButton® and Tidbit® temperature loggers	Potosi, Camp Four, Sunbeam (reference)	Three locations along Potosi and Camp Four, two along Sunbeam	One‐hour interval. Early July–late September (84 days)	Before debris flow 2003–2005. After debris flow 2008–2013
Channel change (profile and valley width)	Light Detection and Ranging (LiDAR)	Potosi, Camp Four	NA	NA	Before debris flow (2002); after debris flow (2011)

We estimated species percent cover using a modified Braun‐Blanquet method (Wikum & Shanholtzer, 1978). Most plants were identified to species, except grasses (Poaceae) were identified to order, rushes (*Juncus*) and sedges (*Carex*) to genera, and mosses (Bryophyta) to division. Wood debris (minimum of 5 cm diameter), water, and bare ground were each recorded as separate categories. Total cover may sum to over 100% owing to overlapping vegetation at different heights. For red alder (*Alnus rubra*) only, we recorded the maximum tree height at each plot. Because the number of plots and transects differed between Camp Four Creek and Potosi Creek, we present the data as the plot mean with one standard error (*SE*).

##### Aquatic insects

We collected benthic macroinvertebrates annually from 2008 to 2012 (late May or June) from one 50‐m reach on each of the debris‐flow streams, Camp Four and Potosi, and the reference stream, Sunbeam (Figure 2). Each stream‐per‐year sample was a composite of ten spatially distributed Surber samples (500‐μm mesh, 0.09‐m^2^ benthos) stored in 80% ethanol. All individuals were counted and identified to the lowest taxonomic level possible, usually genus, although Chironomidae (midge larvae) were identified to subfamily (Merritt et al., 2008). We did not include noninsects (e.g., mites, copepods, ostracods, and worms) in the results because of their ubiquitously low abundance. We assigned each insect taxon a functional feeding group (FFG) that describes the individual's primary feeding mechanism (Merritt et al., 2008). We present insect counts as density (number/m^2^) or proportions (%) of total density. To examine insect community composition and abundance patterns over time, we used nonmetric multidimensional scaling (NMDS, PC‐ORD v.7) with Sorenson distance to ordinate 15 benthic samples by 68 insect taxa with density data (McCune & Grace, 2002). We removed rare taxa that were present in only a single sample (13 rare taxa) and log10(*x* + 1) transformed the densities prior to performing the NMDS. Joint plots (line vectors) display sample‐level taxa richness and density proportions associated with each axis (Pearson's correlation coefficient |*r*| > .5).

##### Aquatic vertebrates and crayfish

Prior to the debris flows, aquatic organism information was available from the REMS study for Potosi and Camp Four creeks, but not the reference stream, Sunbeam Creek. In 2008, we re‐established two 25 m long index survey reaches on Potosi and Camp Four in the same locations where they had been in the REMS study (Figure 2). We located two new 25‐m survey reaches on Sunbeam Creek. The two reaches per stream were labeled “lower” and “upper” relative to their location along the stream. These reaches were approximately 500 m apart along the debris‐flow streams and approximately 200 m apart along Sunbeam Creek. We surveyed each reach in July when stream flow was relatively high and stable, and again in September when flow was low for the year. Before the debris flows, Potosi Creek was surveyed (2002–2006) and Camp Four Creek (2003–2006), and after the debris flows from 2008 to 2012 along with Sunbeam Creek (Table 1). No surveys were done in 2007 because the REMS study had ended and the debris flows had not yet occurred. Sampling consisted of a three‐pass removal electrofishing survey with secure block nets. With each pass, we placed all fish, amphibians, and crayfish into a live bucket, identified them to species, and measured their total length (snout to tip of tail). After the third pass, we removed the block nets and released the animals back into the stream. At each sampling occasion, we recorded reach length and average wetted width and depth.

Species collected were coastal cutthroat trout (*Onchorhynchus clarkii clarkii*), signal crayfish (*Pacifastacus leniusculus*), coastal tailed frog (*Ascaphus truei*), and torrent sculpin (*Cottus rhotheus*, in Potosi and Sunbeam creeks only). For each survey, we calculated species reach densities (number/m^2^) to account for different flow levels between independent sample occurrences. For cutthroat trout only, we first derived population estimates from models based on pass‐depletion trends and then converted estimates to densities and report total lengths (mean, *SD*). We then report age class of the cutthroat trout based on the September data to provide the best snapshot of the young‐of‐the‐year cohorts. Species mean (and *SD*) densities were calculated for each stream and year surveyed, combining July and September surveys and upper and lower index reaches within each stream (*n* = 4). We performed within‐stream before versus after yearly species density comparisons for Potosi Creek and Camp Four Creek with all before‐debris‐flow years grouped as a single control and compared with each after‐debris‐flow year. Comparisons used *t* tests (two‐tailed) assuming unequal variances. We used an analysis of variance (ANOVA) for comparisons between streams (Potosi vs. Camp Four vs. Sunbeam creeks) for each species mean density over five years postdisturbance (2008–2012). Post hoc comparisons of means used Tukey–Kramer Honest Significant Difference (HSD) contrasts, which conservatively adjust the rejection criterion for the number of comparisons. The significance level for tests was *p* ≤ .05.

##### Upstream fish distribution

In the years following the debris flows (2008–2012), we conducted presence/absence electrofishing surveys to measure the changing locations of the farthest (i.e., last) upstream trout and sculpin in Camp Four and Potosi creeks, and for trout only on the nearby reference stream, Sunbeam Creek. We started surveys in September 2008 and thereafter in July and September at the same time as the block‐net removal surveys. Fishing was conducted through every habitat unit (i.e., pool, riffle, rapid, cascade) in ~50‐m increments until sculpin and trout were no longer caught as we moved upstream. If there was a barrier above where the last sculpin was caught, we continued fishing upstream for another 100 m to verify that sculpin were absent. If sculpin gradually disappeared, we continued fishing upstream another 200 m, or to a definite barrier (whichever was less) to verify absence. For trout, we continued fishing 400 m upstream to verify that there were no fish, whether there was a perceived barrier or not. In addition to the length and weight of the farthest upstream fish, we collected a GPS coordinate and measured the location with a tape from the nearest mapped vegetation transect reference point (RP) to validate the distance upstream from the Waddell Creek confluence.

## 3 RESULTS

### 3.1 Channel morphology

On Potosi Creek before the debris flow in 2002, the elevation‐based slope change from the Waddell Creek confluence to the toe of the initiating landslide (3,584 m) was 10.6%, but after the debris flow in 2011, the slope was 9.1% over the same distance—a 14% decrease. Likewise, on Camp Four Creek before the debris flow, the average slope from Waddell Creek to the toe of the initiating landslide (2,535 m) was 9.3%, but after the debris flow, the slope was 8.1%—a 13% reduction.

The active channel width increased from before to after the debris flows at all measured cross sections taken at vegetation reference points (RP's) (Figure 2). Along Potosi Creek, more than 100% widening occurred at three locations with one location at 98% (Table 2). On Camp Four Creek, only one cross section had ~100% change; this was at the lowest cross section just upstream from Waddell Creek. Overall, the cumulative percent increase in floodplain width was similar for Potosi and Camp Four creeks, at 66% and 65%, respectively.

**Table 2:** Floodplain width change (m) at debris flow reference points (see Figure 2) from 2002 (before) to 2011 (after) LiDAR bare earth data

Ref. point (RP)	Distance upstream^a^ (m)	Floodplain width change (m)			
		Before	After	Diff.	% change
Potosi Creek					
1	295	57	69	12	21
2	502	33	46	13	39
3	703	19	34	16	85
4	909	16	39	23	142
5	1,112	24	44	20	80
6	1,350	27	29	2	6
7	1,562	20	49	29	140
8	1,785	22	30	9	39
9	2,007	12	35	23	188
10	2,219	14	27	13	98
11	2,401	13	25	12	88
Mean (*SD*)		23 (13)	39 (13)	15	66^b^
Camp Four Creek					
1	188	39	86	47	119
2	377	38	41	3	9
3	583	19	32	13	68
4	820	18	26	8	46
5	1,013	15	26	12	79
6	1,231	15	26	11	76
7	1,452	16	28	12	72
8	1,683	11	17	6	54
9	1,872	13	22	9	65
Mean (*SD*)		21 (10)	34 (20)	13	65^b^

a Distance (m) from Waddell Creek confluence.

b Cumulative percent change from before compared to after debris flows.

### 3.2 Stream temperature

**Figure 3:**
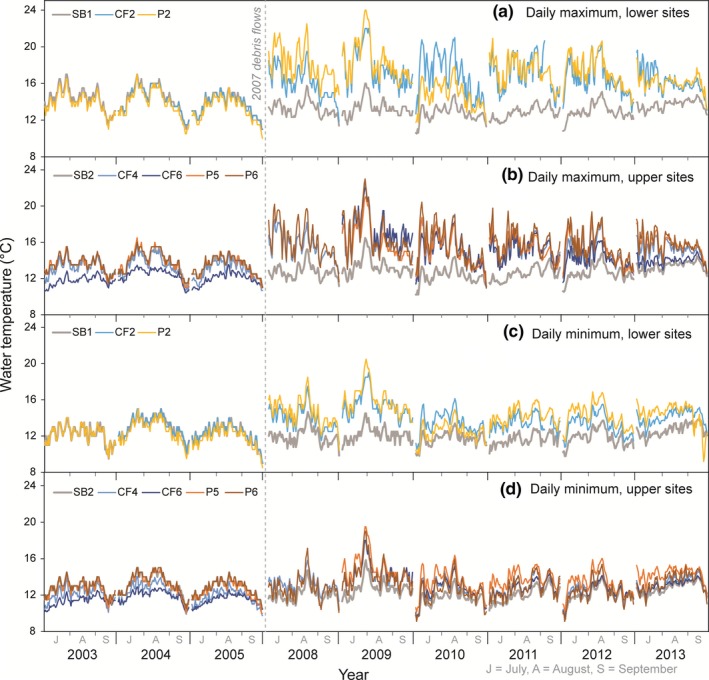
Daily maximum (a & b) and minimum (c & d) water temperatures before (2003*–*2005) and after (2008*–*2013) debris flows at the lower drainage sites (a & c) and the upper drainage sites (b & d) along the reference stream, Sunbeam (SB), and the two streams disturbed by debris flows in December 2007, Camp Four (CF), and Potosi (P)

**Figure 4:**
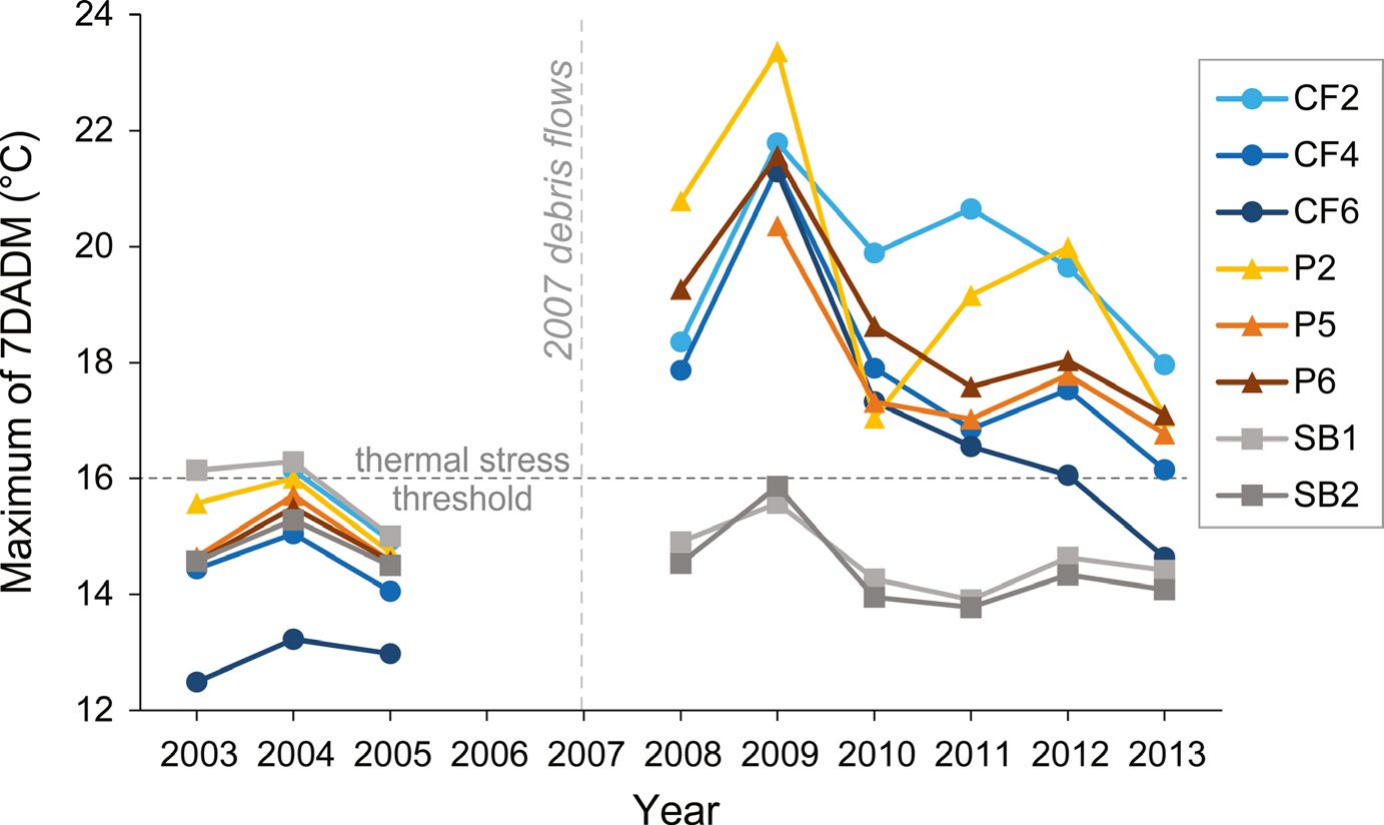
Summer maximum water temperatures of the 7‐day average of the daily maxima (7DADM) before (2003*–*2005) and after (2008*–*2013) debris flows at monitoring sites along the reference stream, Sunbeam Creek (SB), and the two streams disturbed by debris flows in December 2007, Camp Four (CF), and Potosi (P) creeks. The 16°C line marks the EPA (2003) recommended maximum 7DADM criterion for EPA Region 10 salmon and trout juvenile rearing in summer, above which thermal stress may occur

Before the debris flows (2003–2005), summer temperatures in all three streams reflect relatively low daily variability. Lower drainage sites from all three streams (SB1, CF2, and P2) track each other almost exactly for both daily maximum and minimum temperatures (Figure 3a,c). Upper drainage sites (SB2, CF4, CF6, P5, P6) also track each other well, but show some site differences (Figure 3b,d). Specifically, CF6 (the farthest upstream site on Camp Four Creek) exhibited cooler temperatures than the other sites. Overall, predisturbance summer maximum of the 7‐day average of the daily maxima (7DADM) water temperatures per year ranged from 14.5 to 16.3°C at the Sunbeam Creek sites, 12.5 to 16.1°C at Camp Four Creek sites, and 14.6 to 16.0°C at the Potosi Creek sites (Figure 4).

After the debris flows (2008–2013), all sites exhibited large increases in daily variability and maximum temperatures relative to their predebris‐flow condition, as well as to the reference sites on Sunbeam Creek (Figure 3a,b). Daily minimum temperatures also increased at the lower drainage sites (CF2, P2; Figure 3c), but less so at the upper drainage sites (CF4, CF6, P5, P6; Figure 3d). Sunbeam Creek daily minimum and maximum temperatures show no apparent increase or decrease since the storm event in 2007. Overall, postdebris‐flow maximum 7DADM water temperatures per year ranged from 13.8 to 15.9°C at the Sunbeam Creek sites, 14.6 to 21.8°C at Camp Four Creek sites, and 16.8 to 23.4°C at the Potosi Creek sites (Figure 4).

The daily maximum and daily minimum temperature differences for each before‐after‐impact‐control site comparison across all of the postyears were significantly different from zero (*F* test, year *df* = 6, residual *df* = 749, *p* < .0001). The repeated‐measures ANOVA mean estimates were all positive values, evidence for higher temperatures at the debris‐flow site compared with the control site in each of the postyears separately (2008–2013), and compared with the preyears as a group (2003–2005). At the lower drainage sites (CF2 and P2 vs. SB1), from 2008 to 2012, summer daily maximum temperatures increased 4.3°C and minimum temperatures increased 2.3°C on average. At the upper drainage sites (CF4, CF6, P5, P6 vs. SB2), from 2008 to 2012, daily maximum temperatures increased 3.3°C and minimum temperatures increased 1.7°C on average, a little bit less of an increase than sites closer to the mouths of the streams. In 2013, maximum and minimum temperature increases, although still significant, had dampened to an average of 2.5°C and 1.5°C, respectively, most likely due to increased shading provided by sapling red alder trees.

From 2 July to 23 September, the Sunbeam reference sites SB1 and SB2 experienced a total of ten days when water temperatures exceeded the 16.0°C salmonid stress threshold during the preyears 2003–2005, but zero days during the postyears 2008–2013. In comparison, the Camp Four and Potosi sites experienced water temperatures greater than 16.0°C for 0–2 days per year in 2003–2005, but from 2008 to 2013, the lower sites (CF2 and P2) exceeded 16.0°C an average of 49 and 57 days per year, respectively, and the upper sites (CF4, CF6, P5, and P6) exceeded 16.0°C an average of 25, 22, 28, and 35 days/year, respectively. Although stream temperatures increased at all the debris‐flow sites, temperatures exceeded the suggested 23.0°C incipient lethal threshold for salmonids in 2009 on only 6 days at P2 and 1 day at P6 (Figure 3a,b).

### 3.3 Riparian vegetation

**Figure 5:**
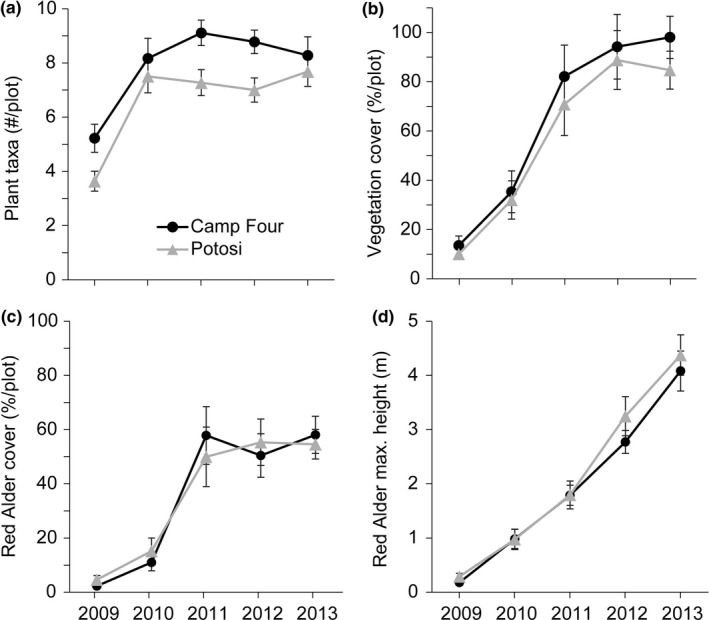
Riparian vegetation metrics (plot mean ± 1 *SE*) along Camp Four Creek and Potosi Creek from 2009–2013; (a) taxa richness, (b) total vegetation % cover, (c) red alder % cover, and (d) red alder maximum height. Debris flows occurred along both streams in December 2007

The mature forest that formed the riparian vegetation was completely removed by the debris flows, as average floodplain width increased 65% along Camp Four Creek and 66% along Potosi Creek (Table 2). In summer 2008, prior to the start of our official surveys, only scattered plant seedlings were observed and the soil was very rocky and unconsolidated. Even in 2009, few plants with little cover were recorded, but colonization and growth increased each year. Taxa richness, on average, was slightly higher in Camp Four Creek than Potosi Creek, although both streams followed the same temporal pattern postdebris flows: low in 2009, a nearly 2‐fold increase in 2010, and then fairly consistent taxa counts through 2013 (Figure 5a). A total of 48 plant species were observed: 2 mosses, 23 forbs, 3 graminoids, 5 ferns, 8 shrubs, and 7 trees (Table A1). Most of the plants were of native origin, whereas only four were known to be introduced species.

Total vegetation cover after the debris flows was initially very low along both streams and then increased each year (Figure 5b). In 2009 and 2010, the plots in both streams were primarily bare ground (55%–79% mean cover), whereas in 2011–2013, the plots were mostly covered by trees (53%–70% mean cover) but also had a scattering of grass, forbs, ferns, and shrubs. The most frequently detected plants were trees: red alder (199 plots), Douglas‐fir seedlings (172 plots), and western hemlock (*Tsuga heterophylla*) seedlings (147 plots). Red alder had the greatest % cover of any plant species at plots along both Camp Four and Potosi creeks. Red alder's mean % cover per plot followed the same temporal pattern as total vegetation, but did not continue to increase between 2011 and 2013 and instead remained around 55% (Figure 5c). The maximum height of red alder steadily increased over time from a mean of 22 cm in 2009 to 423 cm in 2013 (Figure 5d). Camp Four and Potosi creeks differed little in terms of riparian vegetation assemblages and cover amounts. The plant assemblages showed no difference longitudinally along the debris flow track of each stream (i.e., no change with transect location) so that similar communities were found in the erosional, transitional, and deposition areas of the channel floodplains.

### 3.4 Aquatic insects

Initially, the debris flows in Camp Four and Potosi creeks reduced benthic insect density and taxa richness considerably, whereas insects exposed to flooding in Sunbeam Creek were less impacted. Over time, the insect communities in all three streams increased in richness, abundance, and became more similar in composition. In total, 81 aquatic insect taxa were recorded from Camp Four, Potosi, and Sunbeam from 2008 to 2012 (Table A2). However, had chironomids been identified beyond subfamily, many more Dipteran species would have been counted. Mayflies (Ephemeroptera), stoneflies (Plecoptera), and caddisflies (Trichoptera), collectively termed EPT's, made up the majority of taxa richness (range of 70%–88% richness).

**Figure 6:**
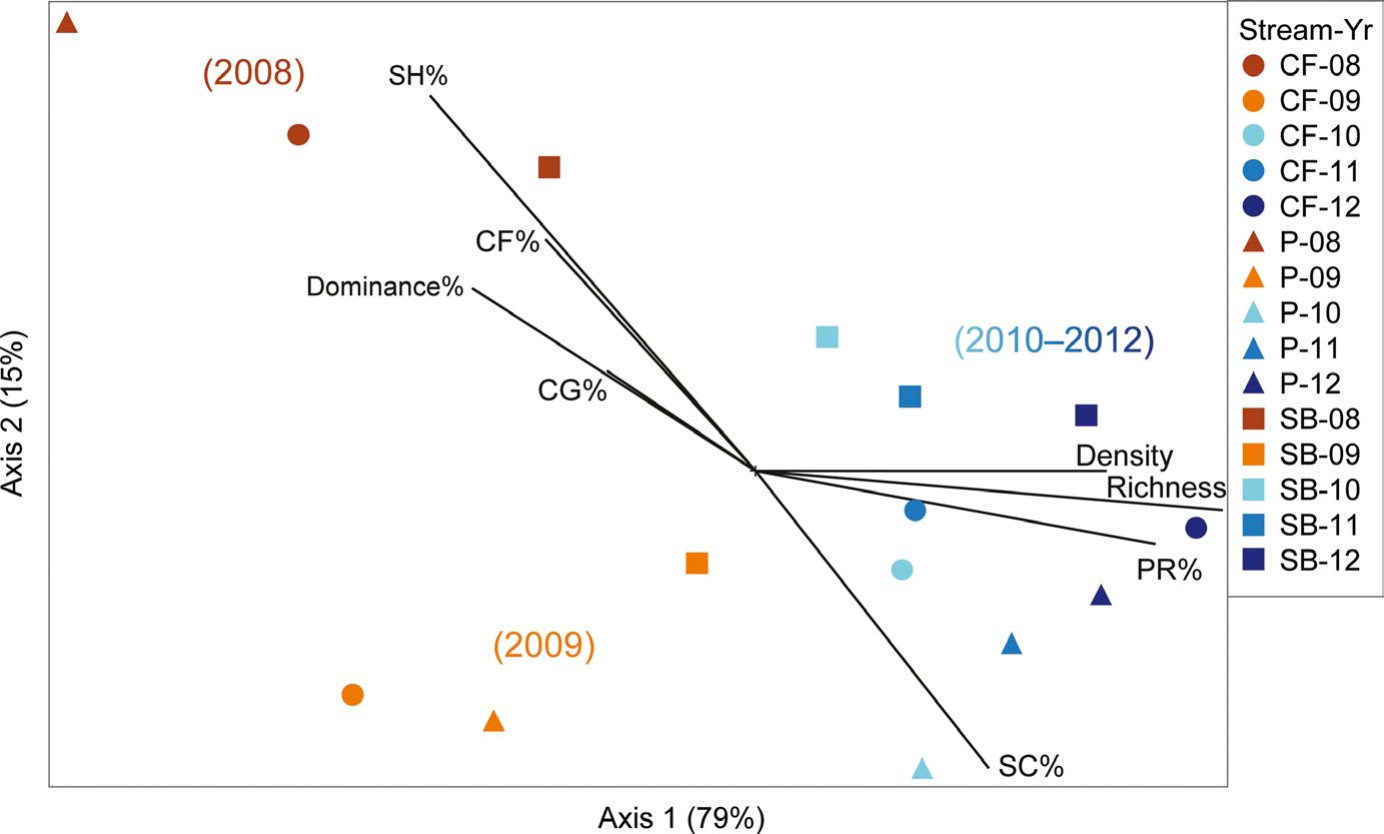
NMDS 2D ordination of 15 benthic insect samples from Camp Four (CF), Potosi (P), and Sunbeam (SB) creeks over five years (2008–2012). Axis 1 explained 79% and axis 2 explained 15% of the variance in the ordination. Joint plots (black lines) show taxonomic and functional feeding group (FFG) proportions correlated with the axes. FFG are scraping (SC), collector‐gathering (CG), shredding (SH), collector‐filtering (CF), and predatory (PR)

Benthic insect communities from all three streams were primarily influenced by time since disturbance (i.e., sample year) as depicted in the resulting 2‐dimensional NMDS ordination of insect densities (stress = 7.68, total *R* ^2^ = .94; Figure 6). However, the Sunbeam communities were more similar to each other across time than the Camp Four and Potosi communities, evidence that the insect communities from Sunbeam Creek were less influenced by time since disturbance than in the debris‐flow streams. Both insect taxa richness and density were positively correlated with axis 1 (*r* = .92 and *r* = .79, respectively). Taxa richness in 2008 was lower in Camp Four and Potosi creeks (24 and 33 taxa, respectfully) than in Sunbeam Creek (38 taxa), but was similar between streams for each of the following years (30–32 taxa in 2009, 38–41 taxa in 2010, 40–44 taxa in 2011, 48–51 taxa in 2012). Total insect density in 2008 and 2009 was lower in Camp Four and Potosi creeks (mean 1,406 insects/m^2^) than in Sunbeam Creek (mean 1,894 insects/m^2^). From 2010 to 2012, Sunbeam Creek had consistently high densities of insects (mean 3,082 insects/m^2^), whereas Camp Four and Potosi creeks had fluctuating high and low densities (mean 2,601 insects/m^2^).

The insect communities in 2008 showed all three streams were proportionately dominated by collector‐gathering (CG) Chironomidae midges (31%–35% density, primarily *Orthocladiinae* and *Chironominae*) and *Baetis* mayflies (27%–33% density), along with shredding (SH) Nemouridae stoneflies (20%–28% density, primarily *Malenka*). The abundance of these taxa, and relative lack of other taxa, leads to the high % dominance by these top three taxa in the 2008 samples (Figure 6). Collector‐filtering (CF) insects, primarily *Simulium*, were also proportionally abundant in 2008 at Camp Four and Potosi (6%–13% density). Scraping (SC) insects were uncommon in 2008 (<2% density), but abundant in 2009–2012 (11%–44% density) and primarily mayflies (*Epeorus, Ironodes, Cinygmula*, and *Drunella doddsii*). Predatory (PR) insects were relatively low in abundance in 2008 and 2009 from Camp Four and Potosi (<3% density), but then increased each year from 2010 to 2012 (6%–14% density). The proportion of predators in Sunbeam Creek remained fairly constant over time (4%–6% density). Initially, *Rhyacophila* was the most abundant predator taxa, but Perlidae stoneflies (*Calineuria californica, Doroneuria*, and *Hesperoperla pacifica*) were more abundant in the later years. Overall, insect densities in 2011 and 2012 were more evenly distributed among taxa and streams compared to earlier years.

### 3.5 Aquatic vertebrates and crayfish

**Figure 7:**
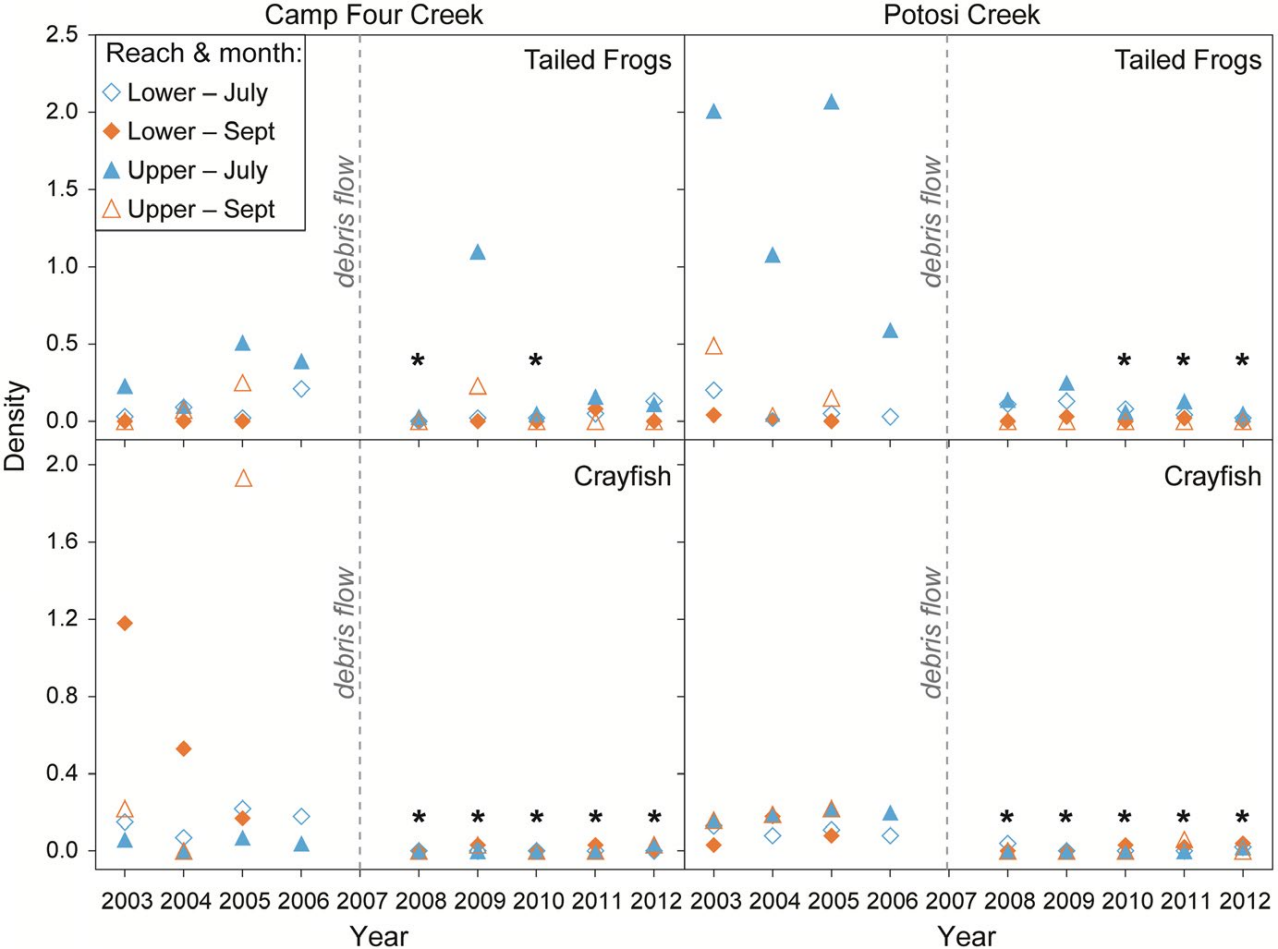
Before and after densities (no./m^2^) of coastal tailed frog tadpoles and signal crayfish occurring on the debris flows. Asterisk mark those years significantly different (*p* ≤ .05) from the combined average of years before the debris flows (Table A3)

Seasonal and spatial variation of tailed frog tadpole density was apparent in both Potosi and Camp Four creeks before the debris flows. Most tailed frog tadpoles were captured in the upper survey reaches in both streams and higher densities always occurred during July surveys both before and after the debris flows. The higher density in July was likely due to metamorphosis of some tadpoles to adult frogs by September. After the debris flow in Potosi Creek, in 2008 and 2009, tailed frog density was not significantly different than before the debris flow, but in 2010 through 2012, tadpole densities were significantly lower than before the debris flow (Figure 7; Table A3). In 2008, only one tailed frog was captured in Camp Four Creek. In 2009, the density increased, but in 2010, the density decreased to a level significantly lower than before the debris flow. In 2011 and 2012, the density increased again in Camp Four Creek and was not significantly different than before the disturbance. Tailed frog tadpole densities after the debris flows showed no significant differences between the disturbed streams and Sunbeam Creek, the reference stream (Table A4).

Signal crayfish densities in both Potosi and Camp Four creeks dropped to nearly zero after the debris flows and did not recover to levels found before the debris flows over five years of monitoring 2008–2012 (Figure 7; Table A3). Crayfish densities in both streams remained significantly lower than the reference stream, Sunbeam Creek (Table A4).

**Figure 8:**
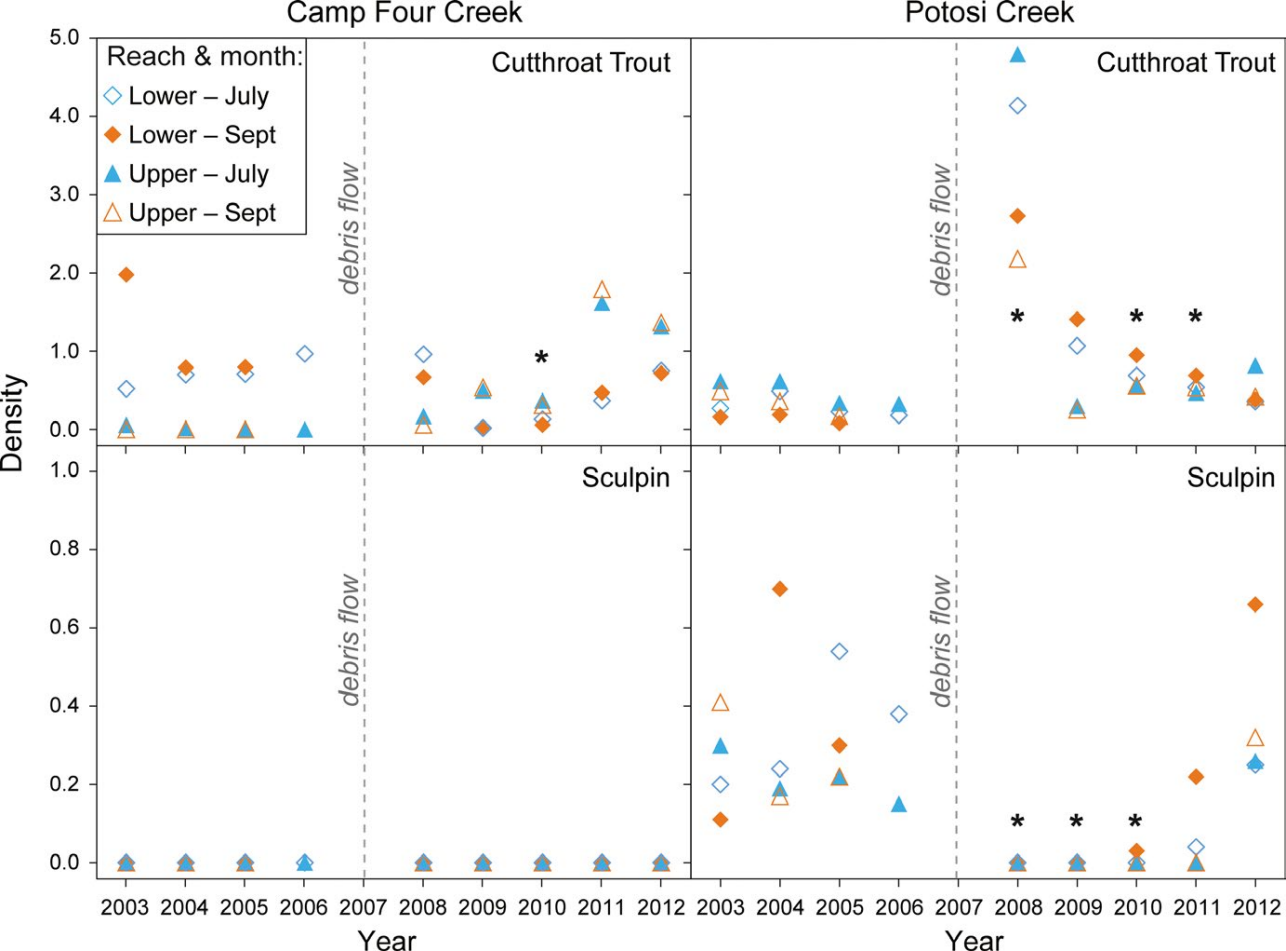
Before and after densities of fish occurring on the debris‐flow streams. Asterisk mark those years significantly different (*p* ≤ .05) from the combined average of years before the debris flows (Table A3). Sculpin do not occur on Camp Four Creek at the survey reaches

Cutthroat trout density in Potosi Creek in 2008, 2010, and 2011 was significantly higher than before the debris flow (Figure 8; Table A3). In comparison, Camp Four Creek trout density in 2008–2009 was somewhat lower than before although not significantly, then became significantly lower in 2010, and rebounded in 2011 and 2012. For the period 2008–2012, Potosi Creek trout density was significantly higher than both the reference stream Sunbeam Creek and Camp Four Creek (Table A4), yet trout densities were not significantly different between Camp Four and Sunbeam creeks. Spatially, the recovery trajectories for trout density differed between the debris‐flow streams. Before the debris flows, trout were found in both upper and lower survey reaches on Potosi Creek, but only in the lower survey reach on Camp Four Creek. After the debris flows, initially cutthroat trout were found in both upper and lower survey reaches of Potosi and Camp Four creeks in 2008; however, starting in 2009, most trout were found in the upper survey reach on Camp Four Creek.

**Figure 9:**
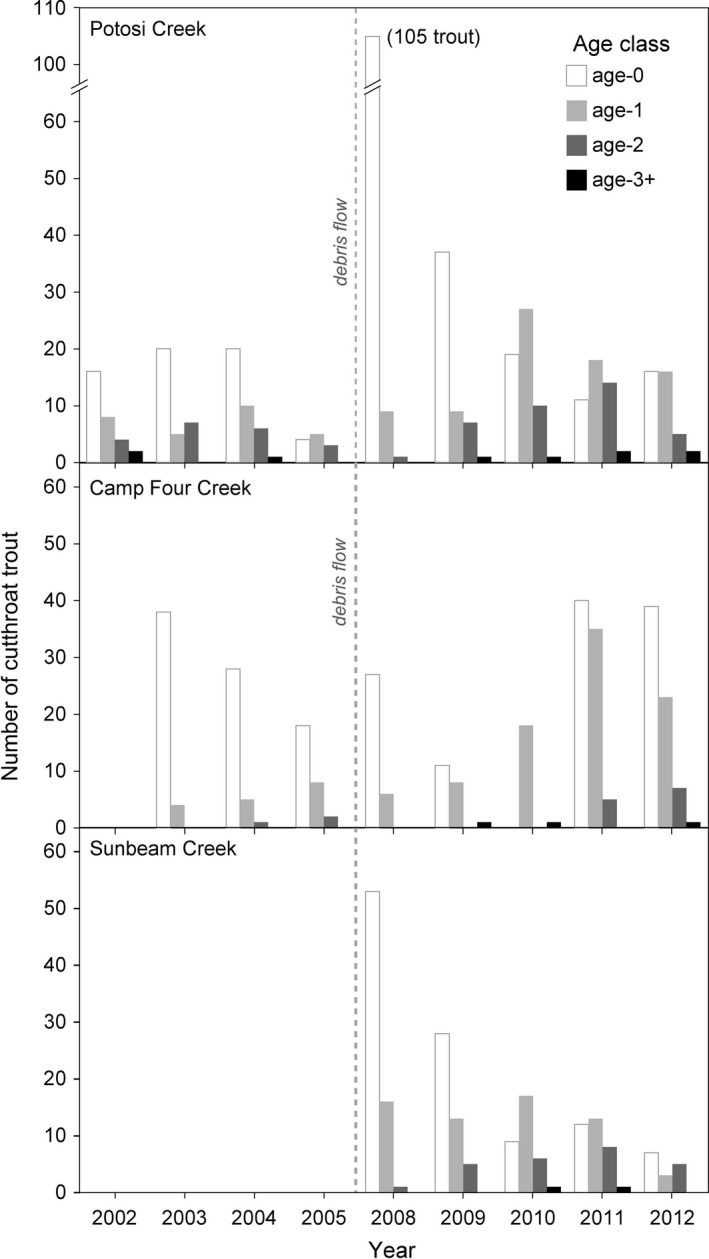
Cutthroat trout abundance by age class and year, from September surveys only. Age classes are based on trout total length: <70 mm for age‐0+, 71–100 mm for age‐1+, 101–130 mm for age‐2+, and >131 mm for age‐3+ fish. Lower and upper survey reaches within each stream were combined. Surveys did not occur in 2002 at Camp Four Creek or before 2008 in Sunbeam Creek (Table A5)

The proportional distribution of cutthroat trout age classes from September surveys on Potosi Creek were fairly consistent in the years before the debris flows (Figure 9; Table A5). Camp Four Creek had more age‐0+ fish than Potosi Creek before the debris flow, but lower proportions of age‐1+ and 2+ fish and no age‐3+ fish observed. In 2008, less than one year after the debris flow, 91% of the trout in Potosi Creek were age‐0+ fish. By 2009, two years after the debris flow, the trout age‐class distribution returned to a state similar to that before the debris flow. Sunbeam Creek also had a high percentage (76%) of age‐0+ fish in 2008, but then decreased in later years. Camp Four Creek also hosted high proportions (86% and 69%) of age‐0+ trout in 2008 and 2009, respectively, but no age‐0+ trout were detected in 2010 when 95% of the trout were age‐1+ fish. By 2012, all four age classes were represented in Camp Four Creek, with an age structure resembling Potosi and Sunbeam creeks.

Sculpins were captured in both lower and upper survey reaches in Potosi Creek before and after the debris flow, and in Sunbeam Creek in 2008–2012. They were never found in either survey reach on Camp Four Creek before or after the debris flow. In Potosi Creek after the debris flow, no sculpins were captured in the reaches in 2008 or 2009, although sculpins were captured much farther downstream during the upstream fish distribution surveys. A single sculpin was captured at the lower survey reach in September of 2010. After that, sculpins were captured in both July and September (2011–2012), and at both reaches (2012). The densities were not significantly different than before the debris flow in 2011 and 2012 (Figure 8; Table A3). Sculpins were found in Sunbeam Creek only in the lower survey reach in 2008 and 2009, whereas they were found in both reaches from 2010 through 2012. Even with these distribution changes in both streams, sculpin densities in Potosi and Sunbeam creeks (2008–2012) were not significantly different from each other (Table A4).

### 3.6 Upstream fish distribution

**Figure 10:**
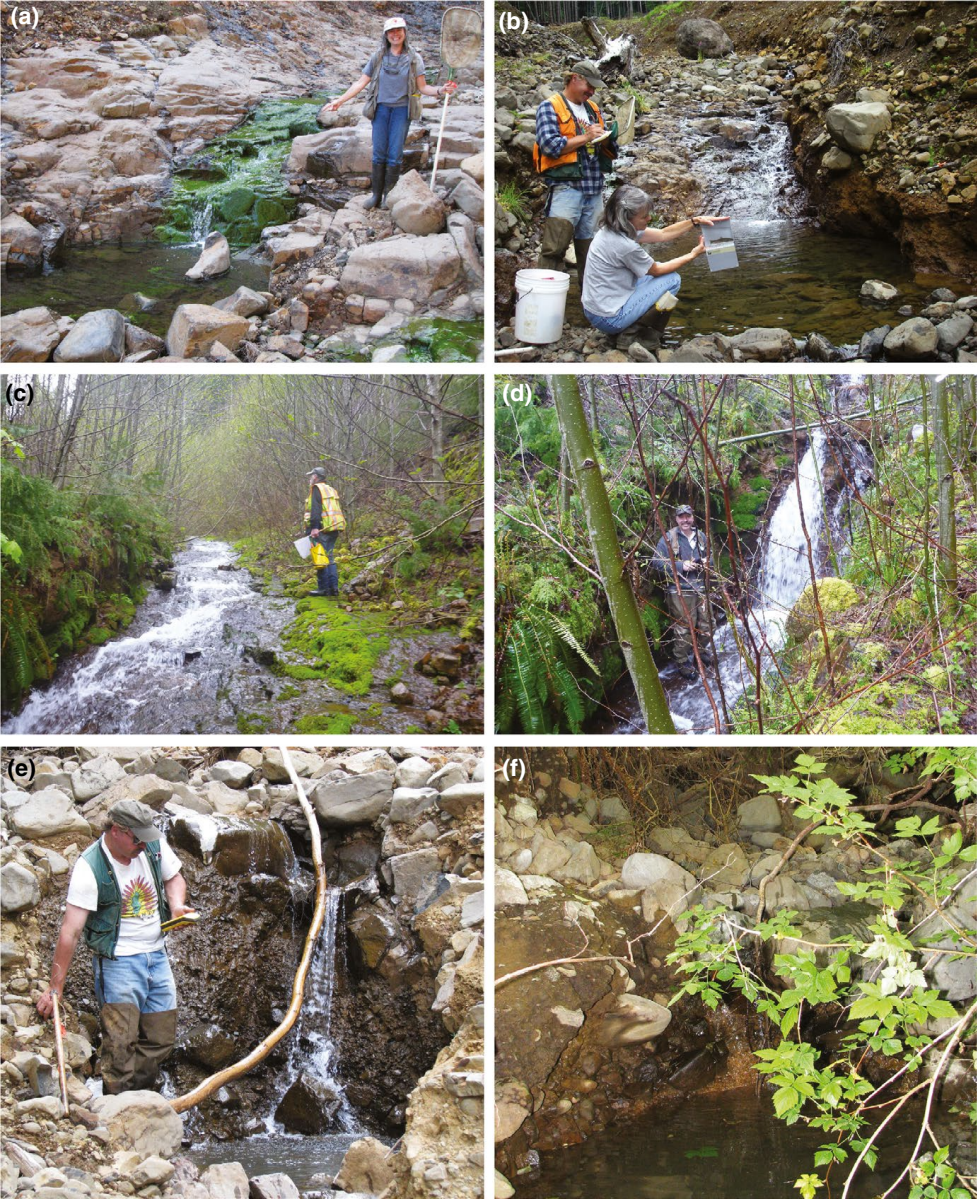
Cutthroat trout upstream barriers. (a) Potosi Creek, Sept. 2008, pool below bedrock chute at 2,427 m (upstream from Waddell Creek); (b) Potosi Creek, July 2009–July 2010, pool below bedrock chute at 2,452 m; (c) Potosi Creek, July 2012; bedrock chute at 2,975 m; (d) Potosi Creek, Sept. 2012, waterfall over bedrock at 2,989 m; (e) Camp Four Creek, 2008–2012, waterfall over unconsolidated material at 1,970 m; and (f) Sunbeam Creek, 2008–2012, pool below wood and sediment dam at 1,613 m

Cutthroat trout were first found in Potosi Creek 2,427 m upstream from the confluence of Waddell Creek in September 2008 and then moved sporadically upstream a distance of 562 m over the following years 2009–2012 (Table A6). Several perceived barriers were surmounted by the trout. In September 2008, trout were found in a pool below a bedrock chute (Figure 10a) and then advanced 25 m upstream to a pool below a 4.9‐m bedrock chute with 34% channel slope that prevented upstream movement from July 2009 through July 2010 (Figure 10b). The most surprising barrier that the trout overcame was a 16.0‐m bedrock chute with 33% channel slope during the period of decreasing flow between July and September 2012 (Figure 10c). The farthest upstream trout in September 2012 was found 2,989 m upstream from Waddell Creek in a plunge pool below a 2.5‐m‐high waterfall over bedrock (Figure 10d). Average stream gradient for 100 m upstream and downstream of this location was 16.1% (*SD* = 13.2; Table A6). Overall, we found that trout movement on Potosi Creek was progressively upstream between survey periods and occurred both during periods of decreasing flows (July–September) and increasing flows (after September).

Cutthroat trout in Camp Four Creek were found 1,970 m upstream of Waddell Creek in September 2008 and remained downstream from a 2‐m waterfall over unconsolidated coarse sediment from 2008 through 2012 (Figure 10e). Between September 2008 and July 2012, a small, steep (30% slope) side channel formed around the waterfall and fish were found in it, facilitating upstream recolonization for an additional 29 m of stream. In September 2012, both the side channel and main channel were dry and fish dropped back downstream 45 m from where they were found in July 2012. Average stream gradient measured at the waterfall was 11.5% (*SD* = 7.0; Table A6).

In Sunbeam Creek, the reference stream, a wood jam with a sediment terrace above it located 1,613 m upstream from Waddell Creek formed a barrier to cutthroat trout from 2008 through 2012. A complex, 2‐m‐high waterfall over the jam was present during July surveys; however, the stream became intermittent as it flowed into the sediment terrace during September surveys to re‐emerge in a pool at the base of the jam where the farthest upstream trout was always found (Figure 10f). Here, the average gradient was 11.0% (*SD* = 7.1; Table A6).

Sculpins in Potosi Creek were first located 339 m upstream from Waddell Creek in September 2008 and then moved upstream another 654 m between September 2008 and July 2009. Sculpins continued to advance upstream through July 2010 to 1,441 m upstream from Waddell Creek, but then retreated downstream 41 m in September 2010 (Table A7). Sculpins were again found progressively upstream in July 2011 through September 2012, where the last sculpin was found in a pool below a 0.5‐m waterfall, 1,891 m upstream from Waddell Creek. Average channel slope at that location was 5.9% (*SD* = 5.2).

Sculpins in Camp Four Creek were more sporadic and inconsistent in their movement upstream than in Potosi Creek. In Camp Four Creek, sculpins were first located 355 m upstream from Waddell Creek in September 2008, but retreated downstream 211 m in July 2009 and then advanced 110 m upstream by September 2009 (Table A7). They continued to move upstream through July 2011, where they were found 907 m upstream from Waddell Creek. However, between July and September 2011 sculpins retreated 472 m downstream, most likely because Camp Four Creek became intermittent above and below the July 2011 location. Between July and September 2012 sculpins advanced upstream 675 m to 1,117 m upstream from Waddell Creek. Average stream gradient at the last sculpin location in 2012 was 6.8% (*SD* = 5.2; Table A7). Sculpin distribution was not monitored in Sunbeam Creek.

## 4 DISCUSSION

### 4.1 Channel morphology

**Figure 11:**
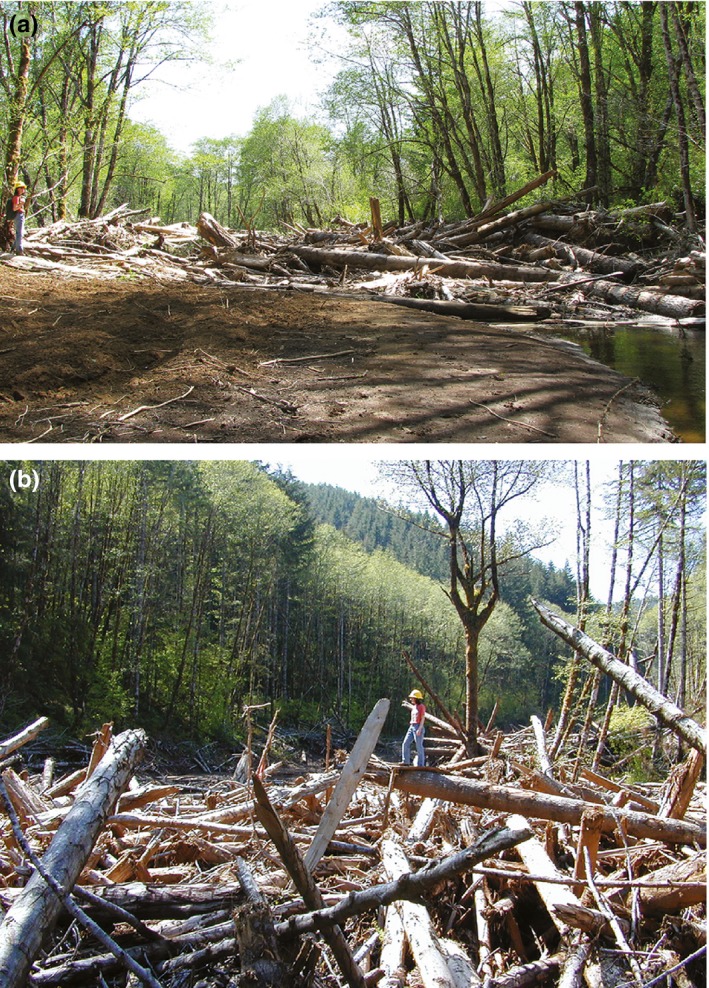
Wood accumulations at the debris‐flow terminus. (a) Potosi Creek confluence, a valley‐spanning log jam along Waddell Creek; (b) Camp Four Creek above the road crossing

Channel slope decreased along the longitudinal profiles of both Potosi and Camp Four creeks. Sediment terraces retained by large logs or accumulations of smaller pieces of wood often act as “steps” and can account for a significant fraction of the total relief along stair‐stepped channels (Lancaster & Grant, 2006; MacFarlane & Wohl, 2003). Debris flows are an effective agent for removing accumulated wood in streams (Hassan, Hogan, et al., 2005). Both wood and associated sediment terraces were removed by the debris flows in this study. In the Potosi Creek debris flow, a large log jam was created in Waddell Creek, extending several hundred meters below the Potosi Creek confluence (Figure 11). The debris flow on Camp Four Creek stopped at the C–8000 road crossing just upstream from Waddell Creek creating a log jam approximately 70 m long at this location.

**Figure 12:**
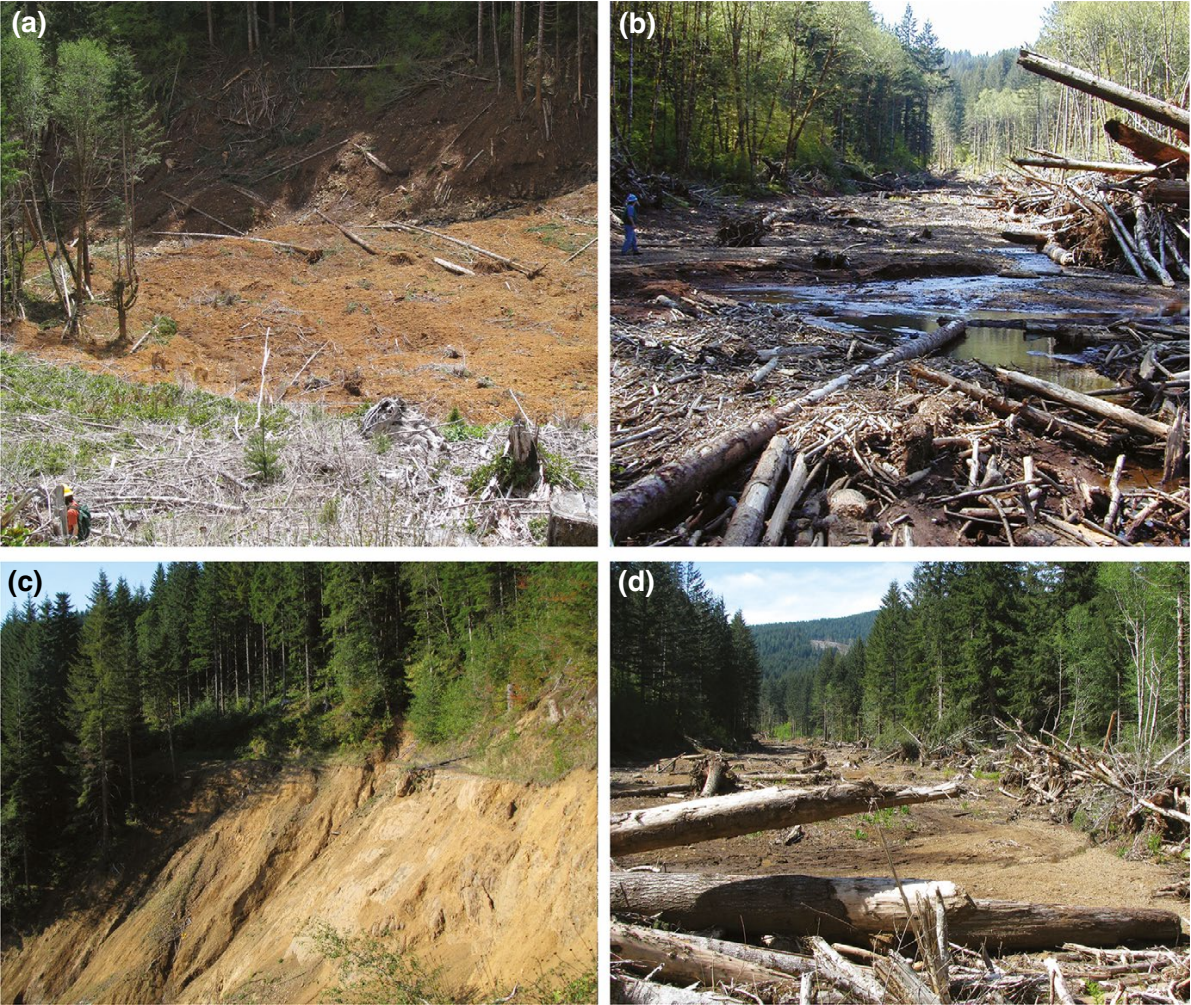
Camp Four Creek debris flow: (a) shallow initiating landslide in a clear‐cut; (b) channel is 22 m wide at 1.9 km downstream from landslide. Potosi Creek debris flow: (c) landslide initiated by road failure; (d) at 3.5 km downstream from the road failure, channel is 69 m wide

The Potosi Creek debris flow initiated from a large road failure at the headwall, whereas the debris flow on Camp Four Creek started from a small landslide in an adjacent clear‐cut. Potosi Creek was scoured to nearly continuous bedrock for about 660 m upstream from RP–11 (Figure 2). In contrast, the debris flow in Camp Four Creek resulted in very little scour to bedrock. This may be due to a lower channel gradient where the initiating shallow landslide entered Camp Four Creek (28%), compared with a steeper gradient on Potosi Creek (40%). Additionally, the landslide on Camp Four Creek entered at an obtuse angle in relation to the channel, whereas the failure at the headwall of Potosi Creek entered the channel at an acute angle and resulted in a significant dam‐break flood when the landslide material was breached during the storm (Figure 12a,c). Increasing scour often occurs when landslides enter channels at acute angles and resulting debris flows from dam‐break floods have longer runout distances (Guthrie et al., 2010). Also, May (2002) found that debris flows initiated at mid‐slope roads, as happened in Potosi Creek, had significantly longer runout lengths and greater total sediment and debris volumes than those from nonroad failures, such as at Camp Four Creek.

In general, the behavior of a debris flow—in particular, how far it travels—depends on the composition of the fluid/debris mixture and on total momentum (Hassan, Church, et al., 2005). Both affected stream channel floodplains experienced substantial widening of their exposed cross sections, averaging a 66% increase in Potosi Creek and a 65% increase in Camp Four Creek. As the debris flows widened the floodplains, they entrained substantial amounts of wood and bank material that became part of the debris‐flow matrix. Channel widening and riparian vegetation removal resulted in a large depositional zone (alluvial fan) in the low‐gradient reach of Potosi Creek upstream from the stream's confluence with Waddell Creek. A similar but much smaller depositional feature formed on Camp Four Creek (Figure 12b,d).

### 4.2 Stream temperature

Stream temperatures increased similarly in both debris flow streams. After six years, temperatures were still higher than before, but less so than initially and more closely resembled the reference stream. The primary cause of warming after the debris flows was increased solar energy from the complete removal of riparian vegetation that provided shade. Solar radiation has been shown to be the largest factor in the stream energy budget on summer days in unshaded streams (Janisch, Wondzell, & Ehinger, 2012; Webb & Zhang, 1997; Wondzell, Diabat, & Haggerty, 2019). Johnson and Jones (2000) found that maximum stream temperatures increased 7°C and high temperatures were seen earlier in the summer when riparian vegetation was removed, either by clear‐cutting and burning or by debris flows. In most cases, stream temperatures gradually return to predisturbance levels after about 15 years, coinciding with canopy closure in the riparian zones regardless of the original cause of vegetation loss. In central Idaho, Welcker (2011) found streams disturbed by recent (<10‐year‐old) debris flows had significantly higher average and maximum stream temperatures, but no change in minimum stream temperatures, whereas streams disturbed by older (~40‐year‐old) debris flows were similar in temperatures compared with nondebris flow reference streams.

Before the debris flows, 80‐year‐old Douglas‐fir trees, with an estimated height of 40 m, were the dominant riparian tree species along Camp Four and Potosi creeks. Daily air temperature variability was moderate, and water temperatures were buffered from changes in air temperatures by shading and reduced air movement provided by the tree canopy. Peak temperatures in the then‐shaded streams occurred in August when stream discharge was low and daytime air temperatures were high. After the debris flows, daily variability increased considerably as water temperatures more rapidly approached air temperatures and maximum water temperatures peaked in July, coinciding with maximum solar inputs. Daily variability and maximum temperatures are likely to moderate as the recovering riparian vegetation grows taller and provides greater shade.

### 4.3 Riparian vegetation

Riparian vegetation was completely obliterated by the debris flows in Camp Four and Potosi creeks but plant recolonization occurred quickly, as found in other case studies. Following a debris flow in the Oregon Coast Range, total vegetative cover increased twofold to sevenfold within three years (Pabst & Spies, 2001). Red alder and salmonberry (*Rubus spectabilis*) were the dominant pioneer species in their study, but by the tenth year they found that rapid growth of red alder allowed it to outcompete salmonberry and inhibit conifer establishment. Our findings were similar, although we found salmonberry to be a minor component of the riparian plant community from the beginning. In our study, the maximum height of red alder increased steadily and rapidly over time, achieving heights greater than 4 m in six years after the debris flows. Seedling conifers were observed along the debris flow tracks adjacent to the streams, but the red alder was clearly outcompeting and suppressing the seedlings. Red alder is an aggressive pioneer species due in part to its nitrogen fixation ability, but is a short‐lived tree (Harrington, 2006); still, we anticipate it will be the dominant tree in the riparian areas for several decades while conifers slowly re‐establish.

### 4.4 Aquatic insects

Aquatic insect populations are often affected by disturbance‐caused changes in food availability or physical condition of stream channels. Most first‐year changes appear to be caused by physical alterations in stream habitat, such as major sediment scouring or deposition, rather than from chemical or thermal changes (Minshall, Royer, & Robinson, 2001). In the first year after a debris flow, benthic insect diversity is typically reduced, with high relative dominance by a few generalist taxa, such as Chironomidae and *Baetis* (Kiffney et al., 2004; Lamberti et al., 1991; Minshall et al., 2001; Mundahl & Hunt, 2011). These insect taxa are considered to be well adapted to disturbance owing to their short generation times and high dispersal abilities via larval drift and as strong adult fliers (Anderson, 1992; Merritt et al., 2008). In Potosi and Camp Four creeks, the densities of Chironomidae and *Baetis* were relatively high 6 months after the debris flows, but other insects (e.g., large‐bodied mayflies, caddisflies, and stoneflies) took multiple years to recover. Even in Sunbeam Creek, which did not have a debris flow, flooding during the storm affected the benthic insect community. Over a period of four years, all three streams steadily increased in richness, abundance, and community similarity.

Removal of riparian vegetation by debris flows increases solar insolation and water temperatures, which support high levels of primary production by algae and large populations of grazing consumers (Cover et al., 2010; Kiffney et al., 2004; Lamberti et al., 1991; Snyder & Johnson, 2006). Less than a year after the debris flows on Potosi and Camp Four creeks, insect scrapers that feed on epilithic algae had extremely low relative abundances, but quickly became the second most abundant functional feeding group (FFG) a year later and maintained their abundances for another three years in both the debris‐flow‐affected streams and similarly in the reference stream. Although not measured, large patches of filamentous green algae and thick mats of diatoms were observed in both debris‐flow streams over the study years, but were not observed in Sunbeam Creek. Elevated water temperatures in Camp Four and Potosi creeks were likely favorable to algal community development in the exposed channels.

Riparian vegetation along Sunbeam Creek the reference stream remained intact and limited sunlight exposure, but stream discharge during the storm was strong enough to mobilize substrate. Therefore, in all three streams, scrapers appeared to be initially limited by substrate stability, as opposed to light or temperature. Strict adherence to a patterned succession of FFG's has not been observed because physical factors, particularly turbidity, sedimentation, and scouring, have an overriding influence on invertebrate occurrence and most stream invertebrates are not narrow food specialists (Minshall, 2003). In addition, insects drifting into our study reaches from tributaries unaffected by the debris flows could have provided a source for immediate and repeated colonization. Changes in insect communities between our streams over time most likely reflected changes in physical conditions and not a lack of available colonists.

Stream food webs are expected to undergo a shift from autochthonous‐ to allochthonous‐dominated energy sources as the riparian vegetation recovers and a forest canopy shades the stream; however, allochthonous energy pathways may take decades to recover to predisturbance levels (Minshall et al., 2001; Wootton, 2012). In the Klamath Mountains of northern California, large wood, benthic organic matter, and detritivorous stoneflies were all very sparse in streams disturbed by 10‐year‐old debris flows (Cover et al., 2010). In headwater streams in Japan, the reduced abundance of shredders was attributed to the loss of large wood and channel structure from debris flows more than ten years prior (Kobayashi, Gomi, Sidle, & Takemon, 2010). After five years, we found the riparian vegetation along the debris flow tracks had grown considerably, but based on the high abundances of scraping insects and low abundances of shredders, it appears that these streams were still primarily supported by autochthonous energy sources.

### 4.5 Aquatic vertebrates and crayfish

#### 4.5.1 Coastal tailed frogs

Some annual variation was apparent in both debris‐flow streams, but overall tadpole densities were lower in both streams, sometimes significantly lower compared with what they were before the debris flows. Studies of coastal tailed frog tadpoles in streams impacted by scorched tree blowdown following the eruption of Mount St. Helens (Washington, USA) also found densities to vary between sites, but to be relatively abundant four to five years posteruption (Crisafulli, Trippe, Hawkins, & MacMahon, 2005). The authors suggest that abundant periphyton, from increased light, and microhabitats in the streams provided survival and recolonization opportunities for tailed frogs, whose tadpoles scrape algae from substrate. Riparian areas along Potosi and Camp Four creeks that were not affected by the debris flows likely provided refuge for adult tailed frogs, which were seen in and near the streams the first summer following the debris flows. Coastal tailed frogs are strongly associated with late‐seral forests that are typically environments of stable temperatures and substrates (Welsh & Lind, 2002)—both of which were highly altered after the debris flows. For example, the highest 7DADM temperature was 23.4°C in lower Potosi Creek. This temperature was within the incipient lethal temperature range (23.4–24.1°C) in which 50% mortality of larval tailed frogs can occur (Claussen, 1973). Nevertheless, both debris‐flow streams experienced many daily maximum temperatures well above 16.0°C, which most likely stressed and affected the recovery of this cool‐water stenotherm.

#### 4.5.2 Signal crayfish

Common in both Potosi and Camp Four creeks before the debris flows, crayfish were nearly absent five years after the debris flows. Crayfish typically mate in October and eggs are carried under the female's tail until they hatch from late March through June. This species is known to burrow into substrates during unfavorable stream conditions. They mature at two years and usually exhibit 10% to 50% survival from egg to adult (Lewis, 2002). The timing of the December debris flows (potentially wiping out an entire cohort), late maturity, extended egg bearing, and poor dispersal ability may all contribute to this species' vulnerability to debris flows. Burrowing behavior would help keep the species from being dislodged during seasonal and high flows but would offer little protection in a debris flow, in which most stream substrate becomes mobilized. It is unknown whether some crayfish escaped the debris flow by occupying habitats in side tributaries thus potentially providing a source for recolonization. Cover et al. (2010) found that crayfish were common in 40+‐year‐old debris flows in northern California streams but not found in recent 10‐year‐old debris flows.

#### 4.5.3 Coastal cutthroat trout

Differences in the recolonization of cutthroat trout in terms of density and age‐class composition between the two debris‐flow streams were not expected. After the debris flows, we found trout density to be relatively similar for the upper and lower reaches of Potosi Creek, but different in Camp Four Creek where density was initially greater in the lower reach, and later, greater in the upper reach. Trout may have moved from the lower to upper reach in Camp Four Creek due to loss of surface flows in the lower reach, or a migration barrier may have been removed during winter high flows in 2008–2009. Before the debris flow, all cutthroat trout detections in both the summer and autumn periods were located in the lower survey reach of Camp Four Creek most likely due to both flow intermittency and barriers.

Potosi and Camp Four creeks both exhibited an initial increase in age‐0+ cutthroat trout, although the increase was delayed in Camp Four Creek. The temporary increase in young trout was similar to patterns of increased abundance found after a volcanic eruption (Bisson, Crisafulli, Fransen, Lucas, & Hawkins, 2005), forest clear‐cutting (Bisson & Sedell, 1984; Hawkins, Murphy, Anderson, & Wilzbach, 1983), and wildfires (Bisson et al., 2003), fitting regional trends of recovery in which early postdisturbance conditions favor survival (Bisson et al., 2009). Similar to our study, the density of age‐0+ brook trout exceeded predebris‐flow levels within one year, and adult density exceeded predebris‐flow levels within two and half years on a debris flow stream in Shenandoah National Park, Virginia (Roghair et al., 2002). However, as we observed on Camp Four Creek, an increase in trout abundance can be delayed until colonists arrive and establish in available habitats. For example, in a debris flow stream in the western Oregon Cascade Range, age‐0+ cutthroat trout declined postdisturbance and remained low throughout the first year, yet in the following years densities increased to about double those found upstream from the debris flow (Lamberti et al., 1991). Similarly, in debris‐flow streams in northern California, steelhead/rainbow trout (*Oncorhynchus mykiss*) biomass initially did not differ from nondebris flow streams, but in subsequent years, trout biomass exceeded that in the nondebris flow streams (White & Harvey, 2017). Following a debris flow in a stream on Washington's Olympic Peninsula, Scarlett and Cederholm (1996) recorded a 50% reduction in cutthroat trout density relative to predisturbance levels. They reasoned that in addition to direct mortality caused by the event, a reduction in total wetted stream area and reduced pool depths after the debris flow were influencing recovery. Colonists from side‐tributary streams, in addition to fish moving upstream from Waddell Creek, could explain the initial abundance of age‐0+ fish in Potosi Creek just 6 months after the disturbance. Some trout may have survived in one or more side tributaries along Potosi Creek, but recolonization of Camp Four Creek likely occurred exclusively upstream from Waddell Creek. Cover et al. (2010) found that trout abundance varied considerably after debris flows in northern California, and showed no consistent recovery pattern; our observations were similar. We found that, at least during initial trout colonization after the debris flows, recovery patterns were site‐specific.

#### 4.5.4 Sculpins

Very little information exists describing sculpin colonization after a debris flow or flood large enough to mobilize the streambed. In Oregon, on two debris flows triggered by a large storm event in 1996, Danehy et al. (2012) found that sculpins repopulated previously occupied areas in about six years. Swanson, Johnson, Gregory, and Acker (1998) found that sculpin densities declined 70% on average from floods caused by the same 1996 storm event in the Cascade Range, Oregon. They also posited recovery times for sculpins to be longer than five years, based on weak dispersal rates and low fecundity. Hawkins and Sedell (1990) found that sculpin populations in streams affected by the 1980 eruption of Mount St. Helens reached population levels comparable to nearby undisturbed streams five years after the eruption. They attributed this rapid colonization to the presence of localized refugia in the streams that provided a source for recolonization. Sculpin densities in Potosi Creek (debris flow) and Sunbeam Creek (flood‐only) were not significantly different; however, sculpin density in Potosi Creek was significantly lower than before the debris flow for three years. In contrast to trout colonization, we believe there were no refuge or source areas for sculpins along Potosi Creek. The side tributaries were too steep for sculpins to occupy (>10% gradient), leading us to believe that sculpins moved upstream from Waddell Creek. Based on the movements and distribution of sculpins in our study streams, full recolonization of previously occupied habitats after debris flows can take several years.

### 4.6 Upstream fish distribution

The farthest upstream habitat occupied by cutthroat trout in each study stream was a plunge pool below a waterfall caused by either a bedrock ledge, large boulders, or a wood jam. These steps along the longitudinal profile can persist for several decades or longer (Hassan, Church, et al., 2005) depending on the longevity and stability of the boulders or logs, and the interval between large, mobilizing flows. Cover et al. (2010) found that streams with recent debris flows had fewer steps along their profiles. In streams with older debris flows, pools were formed by alluvial steps, whereas in streams with recent debris flows, pools were formed by very large boulders or exposed bedrock ledges. Our observations in this study were similar. In general, the debris flows appeared to even the gradient and eliminate rock steps and large wood jams, thus opening up new habitats for trout colonization.

We found the length of the last upstream trout was always greater than 130 mm, representative of an age‐3+ fish. This may be due to size‐structured dominance in salmonid populations (Ward, Webster, & Hart, 2006) and the superior swimming speed and jumping ability of the larger trout. Older trout were the last upstream fish regardless of year‐to‐year changes in age‐class distributions in downstream survey reaches of Potosi, Camp Four, and Sunbeam creeks. Trotter (1989) noted that headwater stream‐resident cutthroat trout become sexually mature as early as age‐2+, but seldom live beyond 4 or 5 years, and exhibit limited instream movement from their birthplace (<200 m). Our results suggest that after debris flows, factors such as cooler water upstream may have influenced longer instream movement by older cutthroat trout.

To our knowledge, recolonization by sculpins has not been monitored where streams have been altered by a debris flow. We found sculpin colonization of the debris flow streams slower and more erratic than that of trout. Before the debris flow, sculpins were present in both the lower and upper survey reaches of Potosi Creek where channel‐slope gradient was 5% and 8%, respectively. They were not found in the lower and upper survey reaches of Camp Four Creek where channel slope was 9% and 11%, respectively. It is possible that sculpins occurred in Camp Four Creek just above its confluence with Waddell Creek before the debris flow, but they did not extend into the lower survey reach at that time. Sculpin recolonization of Potosi Creek after the debris flow continued progressively upstream between survey periods, whereas in Camp Four Creek, sculpin recolonization was more sporadic. Steeper channel gradient, winter high flows, and summer flow intermittency were probably contributing factors to sculpin colonization in Camp Four Creek. Based on the average stream gradient at the farthest upstream sculpin locations September 2012 and in Potosi Creek before the debris flow, we suspect that 6%–10% channel slope may be a threshold for sculpin movement in these small, headwater streams.

## 5 APPLICABILITY OF FINDINGS TO OTHER AREAS

Many of the biological responses to debris flows in our study streams are explained by the interaction between the ecological adaptations of pioneering species and the conditions in the debris flow track. We expect that the pioneer aquatic and riparian species that we observed, common to the Pacific Northwest region, will play analogous roles in the biological responses to debris flows in headwater streams in other areas. However, specific recovery patterns of these species will strongly depend on an array of site‐specific conditions, including the characteristics and timing of the debris flow, refuge locations, weather patterns, and persistence of legacy habitats. To reoccupy a stream after a debris flow, pioneer species need source populations and access routes. Debris‐flow volume, momentum, and the geometry will shape the longitudinal profile and cross section of the headwater valley, all of which we found can affect recolonization.

One of our key findings was how rapidly stream food webs recovered after the severe disturbance created by the debris flows. A food web containing multiple trophic levels supported populations of pioneer species approaching predisturbance levels less than a year after most ecosystem structure and function had been almost completely obliterated. We suggest that pioneer species higher in the food web, like trout, are uniquely adapted for colonizing disturbed streams owing to their strong swimming ability, but to persist after arriving, these species need suitable habitat and food availability. Thus, a key to the success of trout and other large predators for re‐occupying streams after a severe disturbance is the rapid recovery of the aquatic insects. For example, the rapid growth of Chironomidae and *Baetis* insect populations in the debris‐flow streams in the early phase of recovery was likely due to their affinity to drift (from upstream or tributary source populations), fast reproduction (multivoltine), and abundant food sources (legacy organic matter or new periphyton growth). If these insect generalists and their food sources are available in streams shortly after disturbance, higher trophic species may also recover rapidly assuming local conditions permit accessibility.

Often referenced by such terms as “severe” or “catastrophic,” natural disturbances such as debris flows in small watersheds are thought to be important to the long‐term productivity and biological diversity of these and downstream ecosystems (Bisson et al., 2009). These infrequent events are widespread across forested landscapes (Benda, 1990; Benda et al., 2005) and typically create habitat diversity (Bilby, Reeves, & Dolloff, 2003). Long‐term impacts of large disturbance events in a given watershed are influenced by the time for recovery between episodes. Some biota and physical habitats take longer to recover than others, and the rate of recovery appears to be site specific. Given the uniqueness of recovery processes, an increase in the frequency of extreme disturbances may have significant long‐term implications for stream ecosystems if source populations or refuge habitats are eliminated.

The role of disturbance in Pacific Northwest streams, whether caused by debris flows, floods, wildfires, or volcanism, is an important mechanism influencing the structure of aquatic ecosystems (Beechie & Imaki, 2014; Bisson et al., 2003). Species diversity, life history and spatial diversity, and phenotypic plasticity are mechanisms that allow communities and populations to adapt to variable and changing environments. Broadscale disturbances can regulate debris flow activity across landscapes (Benda, 1990; Benda et al., 2005; May & Gresswell, 2003). Given the management regime of Capitol State Forest where our study occurred, we do not know if the frequency of debris flows will be accelerated, remain at natural background levels, or actually decrease over time. We found that rapidly dispersing aquatic organisms quickly recolonized the debris‐flow channels. Other organisms with limited dispersal ability have recovered more slowly; riparian plant communities and aquatic habitats along the streams will likely be altered for decades. Our study continued for only six years following the debris flows, but it is clear that future changes in Potosi and Camp Four creeks can be anticipated, even if we cannot predict exactly what they will be. At present, the short‐term impacts of large disturbances on stream organisms can be reasonably modeled, but long‐term consequences of disturbance and subsequent biological recovery will depend on future climate and management activities.

## CONFLICT OF INTEREST

None declared.

## AUTHOR CONTRIBUTIONS

All authors contributed to the conception and design of the work, or the collection, analysis, and interpretation of data.

## Data Availability

The USDA Forest Service, Pacific Northwest Research Station was the lead sponsor for this work, and related data are publicly available at https://www.fs.usda.gov/pnw/datasets.

## APPENDIX 1

**Table A1:** Riparian plants along Camp Four (CF) and Potosi (P) creeks in Capitol State Forest, Washington, surveyed annually from 2009 to 2013

Life form	Scientific name	Common name	Frequency		Veg % cover	
			CF	P	CF	P
Fern	*Athyrium filix‐femina*	Lady fern	1	0	0.06	0
Fern	*Blechnum spicant*	Deer fern	6	2	0.16	0.05
Fern	*Polystichum munitum*	Western sword fern	6	0	0.22	0
Fern	*Pteridium aquilinum*	Bracken fern	1	2	0.01	0.01
Fern	Polypodiales spp.	Fern	8	6	0.26	0.07
Forb	*Aster* spp.	Asters	4	4	0.04	0.02
Forb	*Adenocaulon bicolor*	Trailplant	3	5	0.02	0.05
Forb	*Anaphalis margaritacea*	Pearly everlasting	5	14	0.07	0.15
Forb	*Asarum caudatum*	Wild ginger	1	1	0.01	0
Forb	*Chamerion augustifolium*	Fireweed	0	3	0	0.05
Forb	*Circium arvense*	Canada thistle	2	2	0.02	0.15
Forb	*Claytonia siberica*	Siberian miner's lettuce	17	13	0.55	0.12
Forb	*Conyza canadensis*	Canadian horseweed	3	3	0.03	0.16
Forb	*Digitalis purpurea*	Foxglove	46	24	0.92	0.39
Forb	*Epilobium ciliatum*	Fringed willowherb	1	0	0.01	0
Forb	*Equisetum* spp.	Horsetail	55	55	8.12	4.99
Forb	*Galium aparine*	Cleavers, stickywilly	8	5	0.08	0.05
Forb	*Heracleum maximum*	Cow parsnip	2	0	0.02	0
Forb	*Lactuca muralis*	Wall lettuce	0	1	0	0.01
Forb	*Leucanthemum vulgare*	Oxeye daisy	2	1	0.06	0
Forb	*Mimulus gutatus*	Monkeyflower	8	3	0.18	0.05
Forb	*Oenanthe sarmentosa*	Water parsley	2	1	0.01	0.02
Forb	*Petasites frigidus*	Arctic sweet coltsfoot	0	21	0	1.3
Forb	*Stachys chamissonis*	Hedgenettle	2	4	0.02	0.03
Forb	*Taraxacum officinale*	Common dandylion	6	7	0.04	0.05
Forb	*Tolmiea menziesii*	Piggy back	23	6	0.64	0.09
Forb	*Veronica serpyllifolia*	Thyme‐leaved speedwell	5	4	0.11	0.03
Forb	unknown	Unknown	18	5	0.36	0.05
Graminoid	*Carex* spp.	Sedge	5	2	0.66	0.02
Graminoid	*Juncus* spp.	Rush	23	18	2.63	0.97
Graminoid	Poales spp.	Grass	50	62	3.57	3.82
Moss	Bryophyta spp.	Moss	14	7	2.41	0.54
Moss	*Lycopodium* spp.	Clubmoss	1	0	0.01	0
Shrub	*Oemleria cerasiformis*	Indian plum	2	0	0.12	0
Shrub	*Rosa* spp.	Roses	2	1	0.02	0.03
Shrub	*Rubus armeniacus*	Himalayan blackberry	0	1	0	0.01
Shrub	*Rubus leucodermis*	Raspberry	2	0	0.02	0
Shrub	*Rubus parviflorus*	Thimbleberry	31	21	0.74	0.61
Shrub	*Rubus spectabilis*	Salmonberry	19	8	0.48	0.2
Shrub	*Rubus ursinus*	Trailing blackberry	10	11	0.16	0.5
Shrub	Unknown	Unknown	1	0	0.01	0
Tree	*Alnus rubra*	Red alder	90	109	34.88	33.98
Tree	*Populus balsamifera*	Black cottonwood	10	14	0.11	0.29
Tree	*Prunus emarginata*	Bitter cherry	0	1	0	0
Tree	*Pseudotsuga menziesii*	Douglas‐fir	74	98	1.13	1.89
Tree	*Salix scouleriana*	Scouler's willow	44	50	1.84	1.98
Tree	*Thuja plicata*	Western redcedar	41	44	1.31	0.69
Tree	*Tsuga heterophylla*	Western hemlock	58	89	1.53	1.92
Wood	na	Wood debris (>5 cm dia.)	58	42	7.54	4.07

Note

Species cover was estimated at 18 plots (5‐m‐diameter circular plots) on Camp Four Creek and 22 plots on Potosi Creek. Species frequency (number of plots with taxa presence) and vegetative % cover (plot mean) are from all five survey years for a total of 90 Camp Four plots and 110 Potosi plots.

**Table A2:** Aquatic insect taxa from Camp Four, Potosi, and Sunbeam creeks in Capitol State Forest, Washington, collected annually from 2008 to 2012

Order/Family	Subfamily/Genus/Species	FFG	Quantity
Coleoptera			
Elmidae	spp. (immature)	Multi	14
	*Heterlimnius*	CG	34
	*Lara*	SH	7
	*Narpus*	CG	2
	*Optioservus*	SC	1
	*Zaitzevia*	CG	10
Staphylinidae	spp.	PR	3
Diptera			
Ceratopogonidae	Ceratopogoninae	PR	22
Chironomidae	Chironominae/Orthocladiinae	CG	6,899
Culicidae	spp.	CG	15
Dixidae	*Dixa*	CG	18
Empididae	spp. (immature)	PR	12
	*Chelifera*	PR	70
	*Clinocera*	PR	186
	*Hemerodromia*	PR	11
	*Neoplasta*	PR	4
	*Oreogeton*	PR	4
Pelecorhynchidae	*Glutops*	PR	7
Psychodidae	spp.	CG	1
Sciomyzidae	spp.	PR	1
Simuliidae	spp. (*Simulium, Prosimulium*)	CF	564
Tipulidae	spp. (immature)	Multi	8
	*Antocha*	CG	6
	*Dicranota*	PR	60
	*Hexatoma*	PR	12
	*Rhabdomastix*	UN	1
	*Tipula*	SH	1
Ephemeroptera	spp. (immature)	Multi	4
Ameletidae	*Ameletus*	SC	57
Baetidae	*Baetis*	CG	8,660
	*Diphetor hageni*	CG	199
Ephemerellidae	*Drunella coloradensis*	PR	120
	*Drunella doddsii*	SC	343
	*Ephemerella (tibialis)*	CG	637
Heptageniidae	spp. (immature)	SC	7
	*Cinygma*	SC	13
	*Cinygmula*	SC	359
	*Epeorus (albertae, longimanus)*	SC	6,029
	*Ironodes*	SC	384
	*Rhithrogena*	SC	50
Leptophlebiidae	*Paraleptophlebia*	CG	895
Plecoptera	spp. (immature)	Multi	17
Capniidae	*Capnia*	SH	1
	*Eucapnopsis*	SH	2
Chloroperlidae	spp. (immature)	PR	38
	*Alloperla fraterna*	PR	2
	*Kathroperla takhoma*	CG	7
	*Suwallia*	PR	75
	*Sweltsa*	PR	264
Leuctridae	spp.	SH	154
	*Moselia infuscata*	SH	4
Nemouridae	spp. (immature)	SH	228
	*Malenka*	SH	2,356
	*Soyedina*	SH	10
	*Zapada (cinctipes, columbiana, frigidia)*	SH	537
Peltoperlidae	*Yoraperla*	SH	184
Perlidae/Perlodidae	spp. (immature)	PR	429
Perlidae	*Calineuria californica*	PR	141
	*Doroneuria*	PR	6
	*Hesperoperla pacifica*	PR	5
Perlodidae	*Isoperla*	PR	3
	*Kogotus*	PR	8
Pteronarcyidae	*Pteronarcys*	SH	55
Trichoptera	spp. (immature)	Multi	23
Brachycentridae	*Amiocentrus aspilus*	CG	1
	*Micrasema*	SH	5
Glossosomatidae	*Glossosoma*	SC	267
Goeridae	*Goeracea*	SC	1
Hydropsychidae	spp. (immature)	CF	135
	*Arctopsyche*	CF	27
	*Hydropsyche*	CF	133
	*Parapsyche*	CF	3
Lepidostomatidae	*Lepidostoma*	SH	114
Limnephilidae	spp. (immature)	SH	2
	*Cryptochia*	SH	1
	*Eccliscosmoecus scylla*	SH	1
	*Psychoglypha*	SH	6
Philopotamidae	*Wormaldia*	CF	22
Polycentropodidae	*Polycentropus*	Multi	15
Rhyacophilidae	*Rhyacophila*	PR	652
Uenoidae	*Neophylax*	SC	202

Note

Taxon quantity (number of individuals) is from benthic samples (10/reach), sum of all five survey years. Taxa in parentheses were identified from a portion of the sample; thus, other species are possibly present. Scientific name to lowest identified taxonomic level, subfamily, genus, or species. Functional feeding groups (FFG) are collector filterers (CF), collector gatherers (CG), predators (PR), scrapers (SC), shredders (SH), and unknown (UN).

**Table A3:** Mean densities (no./m^2^) of cutthroat trout, tailed frog tadpoles, signal crayfish, and sculpins averaged for years before the debris flows (2002–2006) and after the debris flows (2008–2012)

Year	Potosi				Camp Four				Potosi				Camp Four			
	Mean density (no./m^2^)	*SD*	Before versus after		Mean density (no./m^2^)	*SD*	Before versus after		Mean density (no./m^2^)	*SD*	Before versus after		Mean density (no./m^2^)	*SD*	Before versus after	
			*t*	*p*			*t*	*p*			*t*	*p*			*t*	*p*
	Cutthroat trout								Tailed frog							
2002	0.20	0.13			—	—			0.03	*			—	—		
2003	0.39	0.21			0.85	1.00			0.69	0.90			0.13	0.14		
2004	0.42	0.18			0.50	0.42			0.30	0.52			0.09	0.02		
2005	0.21	0.11			0.76	0.06			0.76	1.14			0.20	0.23		
2006	0.26	0.11			0.97	*			0.31	0.40			0.30	0.13		
Combined 2002–2006	0.29	0.15			0.77	0.50			0.42	0.74			0.18	0.13		
2008	2.30	0.55	**−8.75**	**<.01**	0.47	0.42	0.92	.38	0.08	0.06	2.10	.06	0.03	*	—	—
2009	0.76	0.57	−1.55	.22	0.27	0.29	1.91	.08	0.14	0.11	1.70	.10	0.45	0.57	−0.83	.49
2010	0.69	0.18	**−3.78**	**.02**	0.22	0.15	**2.49**	**.03**	0.07	0.01	**2.16**	**.05**	0.03	0.02	**2.75**	**.02**
2011	0.56	0.09	**−4.00**	**<.01**	1.06	0.75	−0.80	.46	0.06	0.06	**2.17**	**.05**	0.09	0.06	1.31	.22
2012	0.50	0.22	−1.60	.19	1.04	0.35	−1.20	.26	0.04	0.02	**2.34**	**.04**	0.12	0.01	1.08	.30
	Crayfish								Sculpin							
2002	0.12	0.15			—	—			0.18	0.10						
2003	0.12	0.06			0.40	0.52			0.20	0.15						
2004	0.16	0.05			0.30	0.33			0.33	0.25						
2005	0.16	0.07			0.48	0.65			0.32	0.15						
2006	0.14	0.08			0.11	0.10			0.27	0.16						
Combined 2003–2006	0.14	0.08			0.32	0.40			0.26	0.16						
2008	0.04	*	—	—	*	*	—	—	*	*	—	—				
2009	*	*	—	—	0.03	*	2.51	**<.01**	*	*	—	—				
2010	0.03	*	—	—	*	*	—	—	0.03	*	—	—				
2011	0.04	0.03	**3.84**	**.03**	*	*	—	—	0.09	0.10	2.54	.06				
2012	0.03	0.01	**6.20**	**<.01**	0.04	0.01	**2.48**	**<.01**	0.21	0.16	0.70	.50				

Note

Bolded *t* statistics and *p*‐values denote significant before (combined years) versus after (each year separate) differences at *p* ≤ .05. Asterisk signifies zero captures or capture only occurring in one sample occasion that year; — = no data. July and September surveys, and upper and lower reach data were combined for each year (*n* = 4). Sculpin do not occur at survey locations on Camp Four Creek, nor was the stream surveyed in 2002.

**Table A4:** Comparison of mean densities (no./m^2^) across sample occurrences between streams affected by debris flows (Potosi and Camp Four creeks) and the reference stream (Sunbeam Creek) from 2008–2012

	*n*	$\bar{x}$	*SE*	95% CI		ANOVA
				Lower	Upper	
*Cutthroat Trout*						
Camp Four Creek	20	0.612	0.141	0.330	0.893	*F_df_*_=2,22.510_ = 5.060
Potosi Creek	20	1.085	0.134	0.816	1.353	*p* = **.010**
Sunbeam Creek	18	0.497	0.148	0.201	0.794	
*Differences statistics for Tukey‐Kramer HSD*						
**Means comparisons**		**Difference**				***p***
Potosi vs. Sunbeam		0.587	0.200	0.107	1.068	**.013**
Potosi vs. Camp Four		0.473	0.194	0.006	0.940	**.050**
Camp Four vs. Sunbeam		0.114	0.204	−0.377	0.606	.841
*Crayfish*						
Camp Four Creek	5	0.032	0.022	−0.012	0.076	*F_df_*_=2,0.070_ = 6.370
Potosi Creek	7	0.033	0.018	−0.005	0.070	*p* = **.010**
Sunbeam Creek	16	0.098	0.012	0.073	0.123	
*Differences statistics for Tukey‐Kramer HSD*						
**Means comparisons**		**Difference**				***p***
Potosi vs. Sunbeam		0.065	0.022	0.011	0.120	**.020**
Potosi vs. Camp Four		0.001	0.028	−0.069	0.071	1.000
Camp Four vs. Sunbeam		0.066	0.025	0.005	0.128	**.033**
*Tailed Frog*						
Camp Four Creek	11	0.180	0.061	0.055	0.305	*F_df_*_=2,1.196_ = 0.754
Potosi Creek	13	0.082	0.056	−0.034	0.197	*p* = .480
Sunbeam Creek	8	0.154	0.072	0.007	0.301	
*Differences statistics for Tukey‐Kramer HSD*						
**Means comparisons**		**Difference**				***p***
Potosi vs. Sunbeam		0.072	0.091	−0.153	0.298	.711
Potosi vs. Camp Four		0.098	0.083	−0.107	0.304	.472
Camp Four vs. Sunbeam		0.026	0.094	−0.207	0.259	.958
*Sculpin (Potosi Creek only)*						
Potosi Creek	11	0.162	0.047	0.067	0.257	*F_df_*_=1,0.740_ = 0.360
Sunbeam Creek	20	0.196	0.033	0.129	0.263	*p *= .555

Subscript for the ANOVA *F_df_* indicates both the degrees of freedom, and error term followed by the *F*‐ratio. Post‐hoc means comparisons (except for sculpins) used Tukey‐Kramer Honest Significant Difference (HSD) contrasts. Significant differences (*p* ≤ .05) are bolded.

**Table A5:** Cutthroat trout length at age

Year	Age‐0+		Mean length [mm] (*SD*)	Age‐1+		Mean length [mm] (*SD*)	Age‐2+		Mean length [mm] (*SD*)	Age‐3+		Mean length [mm] (*SD*)
	*n*	%		*n*	%		*n*	%		*n*	%	
Potosi Creek—before												
2002	16	53	61 (6)	8	27	79 (9)	4	13	113 (6)	2	7	156 (21)
2003	20	63	56 (11)	5	22	80 (7)	7	16	114 (11)	0	0	–
2004	20	54	58 (8)	10	27	84 (9)	6	16	117 (11)	1	3	134
2005	4	42	60 (9)	5	33	82 (11)	3	25	125 (6)	0	0	–
Potosi Creek—after												
2008	105	91	56 (8)	9	8	75 (5)	1	1	115	0	0	–
2009	37	69	60 (7)	9	17	78 (9)	7	13	117 (10)	1	2	132
2010	19	47	61 (4)	27	33	80 (6)	10	18	116 (8)	1	2	162
2011	11	40	65 (4)	18	31	84 (9)	14	24	111 (7)	2	4	137 (4)
2012	16	41	62 (4)	16	41	77 (6)	5	13	108 (5)	2	5	146 (16)
Camp Four Creek—before												
2003	38	90	52 (9)	4	10	80 (8)	0	0	–	0	0	–
2004	28	86	54 (8)	5	12	78 (8)	1	2	103	0	0	–
2005	18	73	54 (8)	8	22	88 (9)	2	5	109 (3)	0	0	–
Camp Four Creek—after												
2008	27	86	63 (4)	6	14	76 (7)	0	0	–	0	0	–
2009	11	69	56 (11)	8	28	79 (6)	0	0	–	1	3	130
2010	0	0	–	18	95	85 (7)	0	0	–	1	5	148
2011	40	50	55 (8)	35	44	77 (6)	5	6	118 (8)	0	0	–
2012	39	56	62 (4)	23	33	80 (10)	7	10	109 (9)	1	1	158
Sunbeam Creek—after												
2008	53	76	57 (8)	16	23	75 (4)	1	1	102	0	0	–
2009	28	61	61 (6)	13	28	82 (10)	5	11	116 (12)	0	0	–
2010	9	27	60 (5)	17	52	82 (8)	6	18	111 (11)	1	3	151
2011	12	35	59 (11)	13	38	78 (9)	8	24	107 (3)	1	3	160
2012	7	47	64 (4)	3	20	86 (10)	5	33	120 (7)	0	0	–

Note

Age‐class separations of <70 mm for age‐0+, 71–100 mm for age‐1+, 101–130 mm for age‐2+, and >131 mm for age‐3+ fish. Showing number (*n*) of trout by age class and % of total trout captured by year (September samples only), mean length (mm) and *SD*. Lower and upper reaches of the streams were combined.

**Table A6:** Farthest upstream cutthroat trout locations on the debris flow and reference streams

Year	Month	Dist. from Waddell^a^ (m)	Dist. moved^a^ (m)	Habitat	Barrier upstream?	Average slope^b^ % (*SD*)
Potosi Creek						
2008	Sept.	2,427	na	Pool—2 m wide, 2.7 m long, 0.38 m deep	Yes—chute over bedrock; 2.7 m long, slope 45%	8.8 (6.5)
2009	July	2,452	25	Pool—3.3 m wide, 4.0 m long, 0.75 m deep	Yes—chute over bedrock; 4.9 m long, slope 34%	8.9 (6.6)
	Sept.	2,452	0	Same as July 2009	Same as July 2009	8.9 (6.6)
2010	July	2,452	0	Same as Sept. 2009	Same as Sept. 2009	8.9 (6.6)
	Sept.	2,649	197	Pool—1.4 m wide, 4.4 m long, 0.35 m deep	No—passable steep cascade	11.1 (9.4)
2011	July	2,683	34	Pool—0.5 m wide, 1.21 m long, 0.4 m deep	Yes—chute over bedrock; 4.0 m long, slope 18%	11.7 (9.5)
	Sept.	2,849	166	Pool—1.1 m wide, 3.2 m long, 0.5 m deep	Yes—chute over bedrock; 3.0 m long, slope 70%	13.6 (10.8)
2012	July	2,975	126	Pool—3.0 m wide, 3.0 m long, 0.6 m deep	Yes—chute over bedrock; 16.0 m long, slope 33%	15.6 (13.1)
	Sept.	2,989	14	Pool—1.0 m wide, 3.0 m long, 0.35 m deep	Yes—vertical falls over bedrock, 2.5 m high	16.1 (13.2)
Camp Four Creek						
2008	Sept.	1,970	na	Pool—2.0 m wide, 2.0 m long, 0.7 m deep	Yes—vertical falls over boulders, 2.0 m high	11.5 (7.0)
2009	July	1,922	−48	Pool in side channel —0.75 m wide, 1.1 m long, 0.13 m deep	Yes—side channel went subsurface in 30 m	11.1 (7.2)
	Sept.	1,928	6	Pool in side channel —0.35 m wide, 0.1 m long, 0.1 m deep	Yes—side channel goes subsurface at pool	11.2 (7.4)
2010	July	1,970	42	Pool—1.6 m wide, 2.2 m long, 0.26 m deep	Yes—vertical falls over boulders, 2 m high	11.5 (7.0)
	Sept.	1,970	0	Pool—1.2 m wide, 1.8 m long, 0.9 m deep)	Yes—vertical falls over boulders, 2 m high	11.5 (7.0)
2011	July	1,970	0	Same as Sept. 2010	Same as Sept. 2010	11.45 (7.0)
	Sept.	1,970	0	Pool—2.1 m wide, 1.8 m long, 0.5 m deep	Yes—side channel goes subsurface in 1 m	11.5 (7.0)
2012	July	1,999	29	Pool in side channel —0.5 m wide, 0.9 m long, 0.1 m deep	Yes—vertical falls over wood, 0.75 m high	11.5 (7.2)
	Sept.	1,954	−45	Pool—0.7 m wide, 1.0 m long, 0.14 m deep	Yes—stream goes subsurface above and below this pool	11.4 (7.0)
Sunbeam Creek						
2008–2012	July/Sept.	1,613	0	Pool—2.5 m wide, 2.0 m long, 0.30 m deep	Yes—falls over jam/sediment dam, ~2.0 m high	11.0 (7.1)

a Measured slope distance from confluence of Waddell Creek.

b Average slope over 200 m (100 m upstream and downstream).

**Table A7:** Farthest upstream sculpin locations on the debris flow streams

Year	Month	Dist. from Waddell^a^ (m)	Dist. moved (m)	Barrier upstream?	Average slope^b^ % (*SD*)
Potosi Creek					
2008	Sept.	339	na	No	3.2 (3.8)
2009	July	993	654	Yes—falls 0.5 m	4.0 (3.7)
	Sept.	1,102	109	Yes—falls 0.25 m	4.1 (3.8)
2010	July	1,441	339	No	5.1 (4.3)
	Sept.	1,400	−41	No	5.2 (4.3)
2011	July	1,579	179	No—short cascade	5.1 (4.4)
	Sept.	1,823	244	No—short cascade	6.0 (5.0)
2012	July	1,851	28	No	5.5 (4.6)
	Sept.	1,891	40	Yes—falls 0.5 m	5.9 (5.2)
Camp Four Creek					
2008	Sept.	355	na	Yes—small chute	3.7 (4.0)
2009	July	144	−211	No	3.1 (4.9)
	Sept.	254	110	No	3.6 (5.5)
2010	July	267	13	Yes—falls 0.5 m	3.5 (5.4)
	Sept.	382	115	No—short cascade	3.9 (4.1)
2011	July	907	525	No—short cascade	6.0 (4.6)
	Sept.	435	−472	Yes—subsurface	4.0 (3.8)
2012	July	442	7	No—short cascade	4.1 (3.8)
	Sept.	1,117	675	Yes—subsurface	6.8 (5.2)

a Measured slope distance from confluence of Waddell Creek.

b Average slope over 200 m (100 m upstream and downstream).
